# JAK-Inhibitors for the Treatment of Rheumatoid Arthritis: A Focus on the Present and an Outlook on the Future

**DOI:** 10.3390/biom10071002

**Published:** 2020-07-05

**Authors:** Jacopo Angelini, Rossella Talotta, Rossana Roncato, Giulia Fornasier, Giorgia Barbiero, Lisa Dal Cin, Serena Brancati, Francesco Scaglione

**Affiliations:** 1 Postgraduate School of Clinical Pharmacology and Toxicology, University of Milan, 20133 Milan, Italy; jacopoangelini1@gmail.com (J.A.); giulia.fornasier@burlo.trieste.it (G.F.); giorgia.barbiero.3@gmail.com (G.B.); lisa.dalcin@unimi.it (L.D.C.); serena.brancati@unimi.it (S.B.); 2 Department of Clinical and Experimental Medicine, Rheumatology Unit, AOU “Gaetano Martino”, University of Messina, 98100 Messina, Italy; 3 Experimental and Clinical Pharmacology Unit, Centro di Riferimento Oncologico di Aviano (CRO), Istituto di Ricovero e Cura a Carattere Scientifico (IRCCS), Pordenone, 33081 Aviano, Italy; rossana.roncato@gmail.com; 4 Pharmacy Unit, IRCCS-Burlo Garofolo di Trieste, 34137 Trieste, Italy; 5 Head of Clinical Pharmacology and Toxicology Unit, Grande Ospedale Metropolitano Niguarda, Department of Oncology and Onco-Hematology, Director of Postgraduate School of Clinical Pharmacology and Toxicology, University of Milan, 20162 Milan, Italy; francesco.scaglione@unimi.it

**Keywords:** Janus kinases, Janus kinase-inhibitors, rheumatoid arthritis, small molecules

## Abstract

Janus kinase inhibitors (JAKi) belong to a new class of oral targeted disease-modifying drugs which have recently revolutionized the therapeutic panorama of rheumatoid arthritis (RA) and other immune-mediated diseases, placing alongside or even replacing conventional and biological drugs. JAKi are characterized by a novel mechanism of action, consisting of the intracellular interruption of the JAK-STAT pathway crucially involved in the immune response. The aim of this narrative review is to globally report the most relevant pharmacological features and clinical outcomes of the developed and incoming JAKi for RA, based on the available preclinical and clinical evidence. A total of 219 papers, including narrative and systematic reviews, randomized controlled trials (RCTs), observational studies, case reports, guidelines, and drug factsheets, were selected. The efficacy and safety profile of both the first generation JAKi (baricitinib and tofacitinib) and the second generation JAKi (upadacitinib, filgotinib, peficitinib, decernotinib and itacitinib) were compared and discussed. Results from RCTs and real-life data are encouraging and outline a rapid onset of the pharmacologic effects, which are maintained during the time. Their efficacy and safety profile are comparable or superior to those of biologic agents and JAKi proved to be efficacious when given as monotherapy. Finally, the manufacturing of JAKi is relatively easier and cheaper than that of biologics, thus increasing the number of compounds being formulated and tested for clinical use.

## 1. Introduction

Rheumatoid arthritis (RA) is a chronic autoimmune disease affecting approximately 0.5–1% of the worldwide population. RA prevalence is higher in women aged between 35 and 50 years than in age-matched men, though this difference is less evident among elderly patients [1,2].

RA is characterized by a chronic synovitis that symmetrically develops in small joints of hands and feet, but any synovial joint can actually be involved [3]. Articular manifestations include swelling, tenderness, warmth, and decreased range of motion. Over time, persistent inflammation leads to the destruction of joints and tendons, and, eventually, to deformities and ankylosis. In some cases, RA can have an extra-articular presentation, and inflammation may involve the skin, heart, lungs, and eyes [4,5]. Generalized malaise and fatigue, pleural involvement, vasculitis, pericarditis, myocardial infarction, rheumatoid nodules, nerve entrapment syndromes, and keratoconjunctivitis sicca are the most common extra-articular manifestations [6]. 

The etiology of RA is multifactorial, and the initial cause is unknown. It is assumed that in a genetically predisposed individual, an environmental agent, like infections or dysbiosis, can trigger an aberrant immune response against self-antigens placed in the articular sites [7,8]. Genetic predisposition is mirrored by the high concordance rates in twins, and several cases have been shown to occur in the same family [9]. More than one hundred genetic loci have been associated with RA risk [10], most of which preside over the control of the immune response [11,12]. Among them, polymorphic variants of the human leukocyte antigen (HLA) genes, coding for molecules involved in the antigen presentation process, have been associated to a more aggressive course of the disease or higher mortality rates [10,13].

Smoking, traumatic events, a low socioeconomic status, educational attainment, and periodontal disease are instead considered environmental risk factors for RA [14,15]. RA may be triggered by gut and oral dysbiosis or by infections sustained by *Proteus mirabilis*, *Escherichia coli*, or the Epstein–Barr virus [16,17]. Epigenetics may play an additional role in RA pathogenesis, being in turn influenced by environmental stimuli [18]. Histone deacetylation, DNA methylation, and microRNA production may affect the transcription of genes involved in inflammation, and are associated with disease risk and activity and response to treatment [19]. 

RA inflammation is due to the clonal expansion of autoreactive T cells, such as T helper (Th)17 lymphocytes and B lymphocytes at the detriment of T regulatory(reg) lymphocytes [20,21]. B lymphocytes mature to the final stage of plasma cells producing autoantibodies, including anti-citrullinated peptide antibodies (ACPAs) and rheumatoid factor (RF) that represent the serologic hallmark of the disease [21]. Autoreactive cells are recruited in the synovial membrane, where resident fibroblast-like and macrophage-like synoviocytes further contribute to the amplification of the inflammatory cascade through the release of several pro-inflammatory cytokines [7], like interleukin (IL)-1 and tumor necrosis factor-alpha (TNF-α), IL-6, and IL-8 [22]. Additionally, Th17 lymphocytes secrete IL-17 that is crucially involved in bone resorption [20]. 

Persistent inflammation results in profound changes of the joint anatomy and physiology, progressing from synovial hyperplasia and endothelial cell activation in the early phase to cartilage destruction and bone erosion in the late phase [23].

The recent 2020 European League Against Rheumatism (EULAR) and the 2015 American College of Rheumatology (ACR) guidelines recommended to start treating RA patients as early as possible owing to the debilitating course of the disease, and subjects should be tightly monitored as therapy should be adjusted according to disease activity [24,25].

The treatment of RA relies on the use of drugs counteracting the aberrant activation of the immune system and includes anti-inflammatory and analgesic drugs, glucocorticoids, disease-modifying anti-rheumatic drugs (DMARDs), immunosuppressive agents, and biologics. The DMARD methotrexate (MTX), and to a lesser extent, leflunomide and sulfasalazine, have been considered as an anchor therapy in RA. Accordingly, their use, as mono- or combo-therapy, is recommended in the early phase of the disease, as well as to treat the milder forms of RA in the long term. Glucocorticoids and non-steroidal anti-inflammatory drugs (NSAIDs) are, instead, indicated for the acute management of RA flares, while the chronic use of these drugs is discouraged. Conventional (c)DMARDs, glucocorticoids, and NSAIDs are characterized by a low target specificity and may unselectively hamper physiological pathways other than the immune response, exposing patients to a not negligible risk of adverse events, like infections or gastrointestinal, cardiovascular, and hematologic disorders [26,27,28]. 

Since their advent in late 1990s [29], biological drugs consisting of monoclonal antibodies or fusion receptors targeting specific molecular or cellular pathways, notably improved the clinical course of RA, allowing the achievement of low disease activity or even remission in a high percentage of cases [30,31]. Additionally, in the last years, the formulation of oral compounds, known as small molecules, able to block some crucial steps of the inflammatory cascade, has further enriched RA therapeutic armamentarium. According to current therapeutic guidelines [24,25], biologic drugs and synthetic small molecules should be prescribed in the case of severe and refractory RA, but these medications may also be considered as the first therapy when poor prognostic factors are present [24].

Among synthetic small molecules, the Janus kinase inhibitors (JAKi) represent a new class of oral drugs counteracting the activation of JAKs, which are cytosolic enzymes presiding over many biologic functions, including the activation of the inflammatory cascade in immune cells [32]. Due to their central role in the immune response and their association with several cytokine receptors, the inhibition of JAKs appeared to be a promising strategy in autoimmune diseases. To date, some oral JAKi (tofacitinib, baricitinib, upadacitinib, peficitinib) have already been licensed for the treatment of RA and other immune-mediated diseases. Results from randomized controlled trials (RCTs) and real-life data are encouraging, and evidence a rapid onset of the pharmacologic effects, which are maintained over the course of time. Their efficacy and safety profile are comparable or superior to those of biologic agents, and JAKi proved to be efficacious when given as monotherapy [33]. In addition, the manufacturing of JAKi is relatively easier and cheaper than that of biologics, and thus noteworthy for increasing the number of compounds being formulated and tested for clinical use. 

The panorama of JAKi designed for RA is extremely innovative and dynamic. The aim of this narrative review is therefore to comprehensively report and compare the pharmacological profile of JAKi approved for the treatment of RA, also providing evidence on JAKi currently under development. We discuss data concerning mechanistic, clinical, and pharmacoeconomic aspects, in order to support clinicians in the identification of the most appropriate place in therapy of these promising new drugs.

## 2. Methods

**Figure 1:**
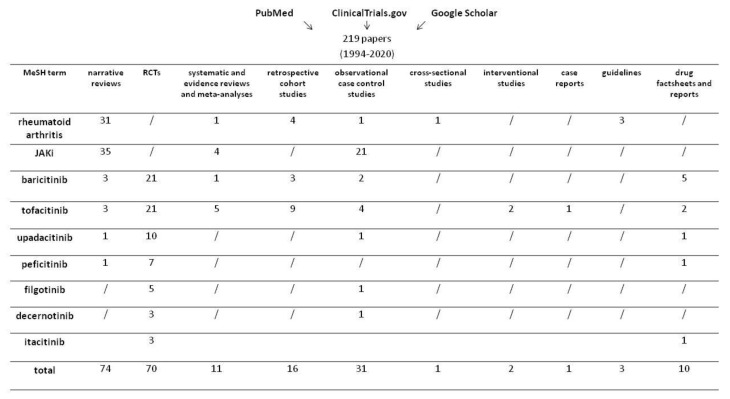
Literature selection process for this article (JAKi, JAK-inhibitors; RCTs, randomized controlled trials).

A literature research was conducted using PubMed, ClinicalTrials.gov, and Google Scholar databases, searching for the medical subject headings (MeSH) terms “rheumatoid arthritis”, “JAK-inhibitors”, “baricitinib”, “tofacitinib”, “upadacitinib”, “peficitinib”, “filgotinib”, “decernotinib”, and “itacitinib”. A total of 219 papers, written in English and published between 1994 and 2020 and pertinent to the purpose of the review, were selected. They included narrative reviews (n. 74), RCTs (n. 70), systematic and evidence reviews and meta-analyses (n. 11), retrospective cohort studies (n. 16), observational case control studies (n. 31), cross-sectional and interventional studies (n. 1 and n. 2, respectively), case reports (n. 1), guidelines (n. 3), and drug factsheets and reports (n. 10) (Figure 1). Results are presented and discussed in the next sections. 

## 3. General Pharmacological Properties of JAKi

Cytokine signaling has been considered as an optimal pharmacological target for the treatment of RA and other autoimmune diseases. Both conventional and biological drugs approved for RA, in fact, act by preventing cytokine release and signaling. 

JAKi block the specific adenosine triphosphate (ATP)-binding pocket, interrupting the JAK-Signal Transducer and Activator of Transcription (STAT) intracellular signaling cascade that ultimately leads to the activation of immune cells and their transition towards a pro-inflammatory phenotype [34]. Four different types of JAKs (JAK1, JAK2, JAK3, and tyrosine kinase 2, TYK2) and seven different STATs (STAT1, STAT2, STAT3, STAT4, STAT5A, STAT5B, STAT6) can variously combine and give raise to different biologic cascades [35], Table 1. JAKs are enzymes belonging to the family of tyrosine kinases constitutively bound to the intracellular domains of type I and II receptors. Type I receptors bind several ILs, colony stimulating factors, and hormones such as erythropoietin, prolactin, and growth hormone. Type II receptors bind interferons and IL-10 related cytokines [36]. 

**Figure 2:**
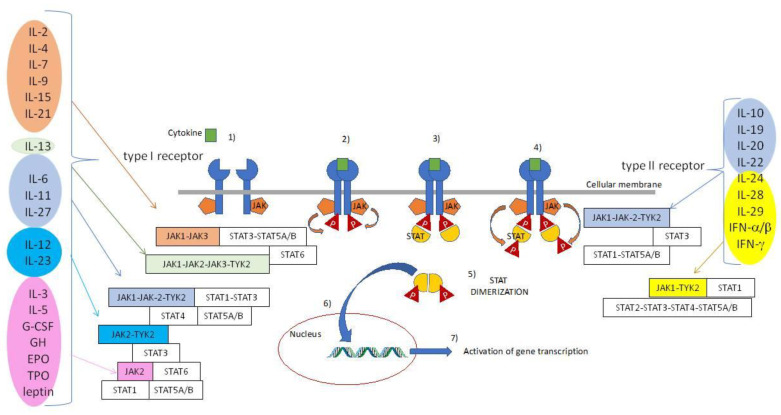
The JAK-STAT canonical signaling pathway. The specific cytokine binds to the transmembrane receptors type I o II. This binding causes the auto-phosphorylation of the receptor itself which summons cytosolic monomeric JAK proteins (**1**). Once recruited, JAKs phosphorylate the receptor (**2**), allowing, in turn, a second phosphorylation of STAT monomers (**3**,**4**). STATs dimerize (**5**) and translocate into the nucleus (**6**), activating the transcription of pro-inflammatory genes (**7**). (JAK: Janus kinase; STAT: JAK signal transducer and activator of transcription; IL: interleukin; G-CSF: granulocyte-colony-stimulating factor; TYK: tyrosine kinase; GH: growth hormone; EPO: erythropoietin; TPO: thrombopoietin; IFN: interferon).

In the canonical pathway, the interaction between cytokines and their cognate receptors induce their dimerization and the downstream activation of JAKs. Generally, cytokine receptors do not have intrinsic kinase activity, and use the binding of JAK enzymes for the autophosphorylation of tyrosine residues present in the cytoplasmic domains. This phosphorylation leads to the docking and the activation of the STAT proteins. JAKs phosphorylate STAT proteins with the consequent dimerization of STAT monomers and translocation into the nucleus. Here, STAT molecules behave as transcriptional factors, promoting the transcription of target genes [37], as shown in Figure 2.

Of note, JAK1 and JAK2 are found ubiquitously and modulate the expression of many inflammatory and non-inflammatory genes in response to IL-6, IL-23, granulocyte colony-stimulating factor, interferons, erythropoietin and other ligands [38,39,40,41,42,43,44,45,46,47,48,49,50,51,52,53,54,55,56,57,59,60,61,62,63,64,65,66,67,68,69]. JAK3 is, instead, expressed in hematopoietic cells and is involved in the signaling cascades unleashed by IL-2, IL-4, IL-7, IL-9, IL-15, and IL-21 [34,36]. 

The JAK-STAT pathway is highly conserved among species due to its involvement in many physiological processes, including the antimicrobial response, metabolism, cell proliferation and self-renew, and tissue regeneration [70]. The kinase activity is strictly regulated by phosphoprotein phosphatases and ubiquitin ligases that dephosphorylate JAKs or induce the proteasomal degradation of the JAK-receptor complex [71]. Accordingly, JAK and STAT loss-of-function and gain-of-function genetic variants have been associated with immunodeficiency and growth retardation and with cancer and autoimmunity, respectively [72].

Besides the canonical pathway, a non-canonical JAK-STAT signaling mechanism has additionally been described in animal models and human cells [73]. In *Drosophila* and mammalian cells, non-phosphorylated STAT forms may shuttle between cytosol and nucleus and affect the euchromatin/heterochromatin ratio without the engagement with STAT-activated genes [74]. Dimeric or multimeric STATs may form cytosolic molecular platforms recruiting chaperones or other proteins associated to organelles or involved in membrane trafficking [75]. Preclinical experiments showed that STAT3 may non-canonically preside over the integrity of microtubules and mitochondria and that STAT5A and STAT5B may control the normal functioning of the rough endoplasmic reticulum [76]. In nucleus, non-phosphorylated STAT1 and STAT3 molecules may couple with other transcriptional factors, like interferon regulatory factor-1 (IRF1) thus influencing the expression of additional genes [75]. Finally, JAK2 may epigenetically control gene transcription through histone phosphorylation [76]. 

It is worth underlining that the JAK-STAT pathway is not the only mechanism orchestrating the immune response in autoimmunity [77]. Other cytokines, like TNF-α, trigger, in fact, distinct intracellular cascades, mostly converging on the activation of the transcriptional factors nuclear factor kappa-light-chain-enhancer of activated B cells (NF-kB) or nuclear factor of activated T-cells (NFAT), which eventually promote the expression of pro-inflammatory genes [78,79]. Notably, these signaling mechanisms may reciprocally influence one another: for instance, it has been shown that NF-kB may induce the expression of the suppressor of cytokine signaling (SOCS)3, in turn inactivating STAT3 in human glioblastoma cells [80], and that NFAT may engage with STAT3 in a dynamic ternary complex promoting the hypertrophy of cardiomyocytes in mouse models [81]. Furthermore, the evidence that JAK and STAT molecules may non-canonically modulate the cell transcriptome without requiring kinase activity should indeed deserve further investigation concerning a presumable residual activity during the pharmacologic inhibition of the JAK-STAT canonical pathway. 

According to their selectivity, JAKi can be divided into first generation JAKi, consisting of non-selective inhibitors, and second generation JAKi, inhibiting the signaling of a narrower range of cytokines. The first generation JAKi encompasses baricitinib, which inhibits JAK1 and JAK2, and tofacitinib, which inhibits JAK1, JAK2, JAK3 and, to a lesser extent, TYK2 [82]; the second generation JAKi, includes, instead, upadacitinib, decernotinib, filgotinib, peficitinib and itacitinib, most of which are still under development. Second generation JAKi seem to have a faster and dose-dependent efficacy and appear more appealing when used as mono-therapy [83]. More selective JAKi should have a better safety profile; however, the complex interplay among cytokines and the ubiquity of the JAK-STAT molecules in cells not belonging to the immune system, though being helpful in the treatment of a broader range of diseases, may increases the risk of unwanted side effects. 

Due to the repression of the immune response, infections, especially of the upper respiratory tract, are the most common side effect during the treatment with JAKi. In addition, reactivation of Herpes Zoster virus (HZV) and alteration in the blood lipid profile have typically been reported under JAKi therapy and appeared to be dose-dependent [84,85,86]. HZV reactivation seems to rely on the repression of the type I interferon response following JAK1 inhibition. Subsequently, vaccination against HZV is recommended before starting JAKi treatment [87,88], especially in some genetically-predisposed ethnic groups and in patients concomitantly prescribed with MTX [89]. JAKi may induce high density lipoprotein (HDL) efflux from macrophages or prevent the IL-6-induced storage of blood lipids into peripheral tissues, and thus increase the level of low density lipoproteins (LDL), HDL and total cholesterol [90], without affecting the LDL/HDL cholesterol ratio. Nevertheless, the increase in blood cholesterol levels has not been correlated to an augmented risk of cardiovascular disease in clinical trials, confirming the theory, also known as “the lipid paradox phenomenon” [91], that in RA cardiovascular morbidity and mortality mostly depend on chronic inflammation rather than on other classical risk factors [92]. Likewise, all clinical studies on JAKi have shown a low incidence of cardiovascular events in treated cohorts of patients probably related to the anti-inflammatory role played by these small molecules [93]. 

Thanks to the interesting efficacy profile emerging from phase III and long-term extension trials [94], it is expected that the use of JAKi for the treatment of RA will notably increase in the next years. This may be further supported by a better therapeutic compliance and a more favorable pharmacoeconomic impact than those of biological agents and their biosimilars. The oral route of administration of JAKi has, in fact, the potential to minimize drug discontinuation in contrast to parentally administered biological products. Finally, the lower manufacturing costs of JAKi compared to those of biologics may result in a more positive pharmacoeconomic trend soon after the expiration of JAKi patent protection. 

In order to provide a comprehensive overview of the panorama of JAKi in RA, the pharmacologic aspects of marketed JAKi and those under development for RA are singularly delineated and discussed in the following paragraphs. 

## 4. Baricitinib

### 4.1. Chemical Structure, Pharmacokinetics, Pharmacodynamics and Mechanism of Action

Baricitinib (International Union of Pure and Applied Chemistry (IUPAC) name: 2-[1-ethylsulfonyl-3-[4-(7H-pyrrolo[2 ,3-d]pyrimidin-4-yl)-1-pyrazolyl]-3-azetidinyl]acetonitrile) is a first generation JAKi, currently solely licensed for the treatment of moderate to severe active RA in adult patients who inadequately respond or are intolerant to one or more cDMARDs. Its chemical structure is that of a pyrrolopyrimidine, being insoluble in water and slightly soluble in hydrochloric acid [95,96]. Baricitinib was obtained by modifying the structure of tofacitinib, another first generation ATP-competitive JAKi. Both the drugs target the JH1 tyrosine kinase domain by interacting with the active conformational site of the ATP-binding pocket [97]. This structure is highly conserved among JAK enzymes, and, consequently, the first-generation JAKi unselectively target several JAKs. Baricitinib prevents the activation of both JAK1 and JAK2 molecules with half maximal inhibitory concentration (IC50) values of 5.9 and 5.7, respectively. This results in the inhibition of the expression of IL-6 in a dose-dependent manner [98]. When given to healthy subjects at a daily dose of 4 mg, the inhibition peaks after two hours since administration and lasts for 24 h [85]. Another relevant effect is the inhibition in vitro of osteoclastogenesis and thus subchondral bone erosions, through the down-regulation of receptor activator of nuclear factor-κB ligand (RANKL) in osteoblasts [99].

In adult patients aged <75 years and without known risk factors, baricitinib is orally administered at a dosage of 2 mg once daily. It can be used either in combination with MTX and other cDMARDs in patients partially responding to these medications or as monotherapy in those who discontinue conventional treatment due to intolerance or to the achievement of their treatment target [100]. Elderly subjects and those with a history of recurrent infections or renal function impairment should receive half-daily dose.

Baricitinib pharmacokinetic profiles after single or multiple administrations are comparable in healthy subjects [101]. After absorption, the bioavailability is 79% and, in blood, about 50% of the drug is bound to plasma proteins. In RA patients, the drug is rapidly absorbed with a time to peak (Tmax) of 1.5 h, and a mean half-life of 12.5 h [102]. Meals decrease the intestinal absorption by 14% and maximum serum concentration (Cmax) by 18–29%. The steady state is obtained within 48 h (or 6× half-life) after the first dose. The mean volume of distribution is 76 L after intravenous (i.v.) administration. Baricitinib is metabolized by hepatic enzymes, mainly belonging to the cytochrome P450 cluster (CYP3A4). Accordingly, the co-administration of baricitinib with CYP3A4 inhibitors or inducers should be carefully evaluated case by case. Nevertheless, studies of clinical pharmacology suggest that baricitinib does not require any dose adjustment in case of mild or moderate hepatic impairment, co-administration of proton pump inhibitors (PPI), CYP3A4 inhibitors (e.g., ketoconazole), moderate CYP3A/CYP2C19/CYP2C9 inhibitors (e.g., fluconazole) or strong CYP3A inducers (e.g., rifampicin) [85]. Furthermore, no meaningful interactions have been reported when other CYP3A drug substrates, such as MTX, simvastatin, ethinyl oestradiol, or levonorgestrel were co-administrated [103]. The drug is however contraindicated in patients with severe hepatic impairment [103].

Baricitinib is mostly eliminated by glomerular filtration and kidney tubule active transportation. This is mediated by organic anion transporter 3 (OAT3), glycoprotein-P, breast cancer resistance protein (BCRP), and multidrug and toxin extrusion protein 2 K (MATE-2K). Studies in RA patients with a preserved kidney function reported a renal clearance of baricitinib of 9.42 L/hour, which is slightly lower than that of healthy volunteers (12 L/hour) [101]. Patients with renal failure require a dose adjustment, and the drug is not recommended in patients having a creatinine clearance < 30 mL/minute [101,104]. Additionally, when baricitinib is co-administrated with digoxin, a substrate of glycoprotein-P, no meaningful alterations were detected [103].

### 4.2. Efficacy

The clinical efficacy of baricitinib as mono- or combo-therapy in patients with RA was assessed in a total of 4 randomized, double-blind, placebo-controlled phase III clinical trials: two long-term (52 weeks) active controlled trials (RA-BEGIN [105] and RA-BEAM Study [106]) and two shorter-term (24 weeks) 3-arm randomized placebo-controlled trials (RA-BUILD [107] and RA-BEACON [108]).

Patients who completed one of these main phase III clinical trials or a phase II exploratory trial [109] were eligible to entering the ongoing phase III single-blind multicenter long-term extension study RA-BEYOND [110,111]. These studies recruited RA patients naïve to conventional and biologic drugs (RA-BEGIN), those failing a precedent treatment with at least one cDMARD (RA-BEAM, RA-BUILD), or those failing a previous biologic therapy, including an anti-TNF agent (RA-BEACON). Studies differed in design and statistical analysis. Specifically, the RA-BEGIN trial tested the non-inferiority of baricitinib vs. MTX, while RA-BEAM, RA-BUILD and RA-BEACON were superiority trials vs. placebo. All the registration trials achieved the primary endpoint, consisting of the percentage of patients meeting the ACR20 improvement criteria (ACR20) at week 12 or 24. In addition, all major secondary endpoints, including ACR50 and ACR70 response rates, disease activity score on 28 joints by C-reactive protein (CRP) (DAS28-CRP) response, health assessment questionnaire disability index (HAQ-DI) response, simplified disease activity index (SDAI) remission rate and improvement from baseline in patient reported outcomes (PROs), were reached in the baricitinib arm compared to placebo. Of note, in the RA-BEAM trial [106], baricitinib was head to head compared with the anti-TNF monoclonal antibody adalimumab, resulting in a significant superiority to adalimumab in ACR20, ACR50 and ACR70 response rate until week 52, and in DAS28-CRP scores, SDAI remission achievement, HAQ-DI scores, and several PROs [112] at week 12.

In the RA-BUILD trial [107], a statistically significant reduction in the radiographic progression of structural joint damage from baseline to week 24 was observed for both 2 mg and 4 mg baricitinib groups compared with the placebo, though the effect was stronger with baricitinib 4 mg/day. These results were confirmed in a two-year analysis in patients who completed RA-BEGIN, RA-BEAM, and RA-BUILD trials [111].

The design of RA-BEYOND [110,111] included also a sub-study in which patients who received baricitinib 4 mg once daily for at least 15 months in originating studies and who achieved sustained low disease activity or remission were re-randomized to continue receiving baricitinib 4 mg once daily or stepping down to 2 mg daily with or without cDMARDs. This step-down sub-study was designed in accordance with international therapeutic guidelines, which recommend a dose tapering (but not discontinuation) of DMARDs in patients who have achieved a sustained disease control [25,113]. After 48 weeks, most of the patients assigned to either a step-down strategy or standard regimen were still in low disease activity or remission [114]. These results, together with some safety trends, including adverse events and infection rates that would seem to favor a 2 mg daily dose, suggest that a dose tapering strategy could be considered in those patients whose RA disease activity has been kept under control with an inductive standard regimen. However, compared with the 4 mg group, the reduction to 2 mg was associated with a modest but statistically significant increase in tender and swollen joint count, physician global assessment, DAS28-CRP, clinical disease activity index (CDAI), and SDAI scores.

A synthesis of the main characteristics of the reported clinical trials on baricitinib is provided in Table 2.

### 4.3. Selected Populations

#### 4.3.1. Pediatric Patients

No published data are currently available concerning the use of baricitinib in the pediatric population affected by juvenile idiopathic arthritis (JIA), although three phase III clinical trials (ClinicalTrials.gov ID: NCT03773978, NCT03773965 and NCT04088396) are at present evaluating the efficacy and safety of baricitinib in patients aged from one or two years to less than 18 years and affected by polyarticular or systemic JIA. The achievement of the clinical endpoints in JIA is challenged by the disease’s unpredictable expression and course. JIA encloses polyhedral manifestations, which may variously involve joints (poly- and pauci-articular forms) or extra-articular sites, or have a systemic development with distinct autoantibody patterns [115]. Around two thirds of pediatric patients affected by JIA may progress to other forms of arthritis in adulthood, which include RA but also seronegative arthritis. However, pathogenic pathways in JIA and adult RA are largely overlapping [115], and the use of oral JAKi could be of great interest in this subset of patients, whose compliance to parenterally administrated drugs, like biologics, is often limited.

#### 4.3.2. Selected Ethnic Groups

Due to the differences between Asian and non-Asian populations in terms of genetic background [116], RA prevalence [117], demographic characteristics and clinical practice (patients with RA in Japan are often prescribed with lower doses of MTX compared with patients in the United States, US [118]), additional subgroup analyses of the four main phase III trials were performed in order to evaluate the efficacy and safety profile of baricitinib in 394 Japanese patients and to assess whether results in this ethnic cohort were consistent with those emerged in overall study population [119]. In all phase III RCTs, the safety and tolerability profile of baricitinib in Japanese patients appeared acceptable and generally consistent with results from the prior phase IIb study of baricitinib in Japan [120] and its long-term extension [121], and with the overall study population data. An increased tendency to develop HZV reactivation under baricitinib was however reported in this ethnic group [122].

### 4.4. Safety

The safety profile of baricitinib, emerging from the registration clinical trials and further analyses [105,120,123,124,125,126,127,128] on more than 3400 RA patients receiving a single dose of the drug, showed an incidence rate of serious adverse events (SAE) of eight in 100 patient-years of exposure (PYE) and a mortality rate of 0.33/100 PYE.

The majority of the reported adverse reactions were infections and hypercholesterolemia observed in ≥ 1/10 cases. The most common infections (with a prevalence rate between 1/10 and 1/100) were pneumonia, HZV reactivation, gastroenteritis, urinary tract infections, and cellulitis. Hypercholesterolemia was a dose-dependent event. However, following an increase in the first 12 weeks of treatment, LDL and HDL serum levels were reported to stabilize [128]. No differences in terms of major cardiovascular events (MACE) were recorded between baricitinib and placebo in RCTs [129].

Generally, after 16 weeks of treatment, an increase in alanine transaminase (ALT) and aspartate transaminase (AST) was observed, especially when MTX was administered in combination [77,85,104].

RCTs also evidenced an alteration in the blood count of platelets and hemoglobin blood value. In particular, platelet counts may increase in the first two weeks of therapy and then stabilize. Hemoglobin may initially decrease and then slowly increase [77,104].

Japanese patients were reported to have a higher risk of HZV infection compared to general population [122].

Evidence on long-term safety still relies on the completion of the ongoing long-term extension studies, as well as pharmacovigilance real-life data. The most common adverse drug reactions during baricitinib therapy reported by Medical Dictionary for Regulatory Activities (MedDRA) system organ class in Eudravigilance, Food and Drug Administration (FDA) adverse event reporting system (FAERS), and Vigiacess database, updated until 28 February 2020, are presented in Table 3.

### 4.5. Pharmacoeconomics

According to two pharmacoeconomic studies, baricitinib has been considered a cost-effective treatment for RA patients with a previous inadequate response or intolerance to cDMARD therapy compared to adalimumab [130,131]. The same result was confirmed in the analysis including the hypothetical discount scenario of market entry of adalimumab biosimilar [130]. From a US budget analysis, baricitinib was considered an equally effective and less expensive option compared to other biologic (b)DMARDs in RA patients with an active disease and an inadequate response to previous anti-TNF agents [132].

Although international recommendations did not express a definite ranking, a single technology appraisal from the National Institute for Health and Care Excellence (NICE) estimated an incremental cost-effectiveness ratio (ICER) for baricitinib, in combination with MTX, vs. intensive cDMARDs being £37,420 per quality-adjusted life year gained (QALY). This value is included in the range usually considered by NICE as a cost-effective use of national health service resources. The use of baricitinib in combination with MTX was judged less cost-effective than RTX plus MTX by NICE. Consequently, it was recommended as an option for patients with severe RA who can tolerate MTX if: (1) they have cDMARD inadequate response; (2) they have an anti-TNF inadequate response and rituximab (RTX) in combination with MTX is not an option; or (3) they have an anti-TNF inadequate response and have already been treated with RTX. NICE also recommended baricitinib in monotherapy or in combination with MTX as a cost-effective use of National Health Service resources in patients with severe RA, except for patients with inadequate response to anti-TNF who are RTX-eligible. In the latter case, the ICER for etanercept biosimilars, certolizumab pegol and adalimumab, all in combination with MTX, were lower than £30,000 per QALY compared with baricitinib in combination with MTX [133].

## 5. Tofacitinib

### 5.1. Chemical Structure, Pharmacokinetics, Pharmacodynamics and Mechanism of Action

Tofacitinib (IUPAC name: 3-[(3*R*,4*R*)-4-methyl-3-[methyl(7*H*-pyrrolo[2,3-d]pyrimidin-4-yl)amino]piperidin-1-yl]-3-oxopropanenitrile) is a pyrrolopyrimidine, a N-acylpiperidine, a nitrile and a tertiary amino compound. As citrate salt, it is soluble in dimethyl sulfoxide (DMSO) at a concentration of 100 mg/mL, slightly soluble in water at a concentration of 2.9 mg/mL after warming, and very slightly soluble in 99.5% ethanol [134].

Tofacitinib is a non-selective first generation JAKi. The drug has three labeled indications: RA, psoriatic arthritis (PsA) and ulcerative colitis (UC), and acts by inhibiting JAK1, JAK2, JAK3 and, to a lesser extent, TYK2.

Tofacitinib is orally administered at a dosage of 5 mg twice a day. After oral administration, it is rapidly absorbed with a Tmax of 0.5–1.0 h, and has a half-life of 3.2 h [135]. The drug has a dose-proportional pharmacokinetics. The steady-state is obtained 24–48 h after the first dose. Food decreases Cmax by 32% without affecting the area under the curve (AUC).

The mean volume of distribution is 87 L after i.v. administration, with an equal distribution in red blood cells and plasma. About 40% of the drug is bound to plasma proteins.

Tofacitinib inhibition of STAT is reversible 24 h after the cessation of treatment, while it lasts for 2 weeks in patients who received the treatment for a minimum of 4 consecutive weeks [82]. In ex-vivo experiments, it has been shown that tofacitinib decreases the expression of the IL-6 gene after a treatment period of 12 to 24 weeks, having instead a variable effect on that of IL-8, TNF-α and IL-10 genes [82]. In a study on RA patients, the serum levels of TNF-α, IL-17, IL-6, and IFN-γ significantly decreased following a four-week treatment with tofacitinib, whilst those of IL-35, mirroring Treg lymphocyte response, augmented [136]. Similar results were reported in responder psoriatic patients after 4 weeks of treatment [137]. In addition, in vitro studies exposing synovial membrane samples of PsA patients to the drug, further demonstrated an additional anti-angiogenic and anti-migrational effect [138,139]. Of note, tofacitinib suppresses in vitro the action of antigen presenting cells, by reducing the expression of the costimulatory molecules CD80/CD86 and by preventing the release of type I interferon [140]. Treatment with tofacitinib up to six months has been associated with a dose-dependent effect on white blood cells of RA individuals, including a reduction in natural killer (NK) cells (usually at 8–10 weeks of therapy and with a spontaneous reconstitution within 2–6 weeks after the discontinuation of treatment) and an increase in B cell count [141]. In healthy volunteers, no significant change in T-lymphocyte and their subsets has been reported in the short-medium term, whereas a prolonged treatment (approximately 5 years) has been associated with a reduction in T cells and an increase in NK cells from baseline [142]. Of note, lymphocyte subsets normalize after the temporary discontinuation of treatment and have not been associated with serious or opportunistic infections or with HZV reactivation. Although influencing the count of B cells in RA patients, tofacitinib seems not to affect the production of antibodies in healthy individuals [142]. However, a study conducted on umbilical cord blood B cells and B lymphocytes of tofacitinib-treated patients evidenced that the drug may interfere with the maturation of B naive lymphocytes [143].

Tofacitinib is the only non-selective JAKi able to prevent the tolerogenic IL-27 pathway, in turn mediated by TYK2 signaling [144]. However, the drug is also able to hamper the STAT3-mediated differentiation of Th17 lymphocytes, thus counteracting the development of autoreactive cells [145].

As for baricitinib, tofacitinib has a hepatic metabolism through the cytochrome CYP3A4 [135], and, to a lesser extent, CYP2C19. Accordingly, also in this case, the co-administration of tofacitinib with CYP3A4 and CYP2C9 inhibitors or inducers should be carefully monitored.

Contrary to baricitinib, the excretion of tofacitinib is mainly via the gastro-intestinal apparatus with only 30% of inactive metabolites excreted in urine. The renal and hepatic clearance are 124 mL/min and 289 mL/min, respectively [134,135]. Therefore, the use of tofacitinib appears safer than that of baricitinib in patients with an impairment of the renal function. Tofacitinib can be also used in patients with a moderate hepatic failure, though a dose reduction is required [146].

### 5.2. Efficacy

The clinical efficacy of oral tofacitinib 5 mg and 10 mg twice daily as monotherapy or in combination with cDMARDs for the treatment of RA has been reported in six pivotal randomized, double-blind, multicentric phase III clinical studies (ORAL Solo—A3921045 [147]; ORAL Start—A3921069 [148]; ORAL Sync—A3921046 [149]; ORAL Scan—A3921044 [150]; ORAL Standard—A3921064 [151]; ORAL Step—A3921032 [152]), and in two open-label long-term extension studies (ORAL Sequel Study—A3921024 [87] and the Japanese study A3921041 [153]). Two studies (ORAL Scan and ORAL Start) assessed radiographic outcomes [148,150]. Recruited patients included MTX-naïve subjects (ORAL Start trial), inadequate responders to MTX or other cDMARDs (ORAL Scan, ORAL Sync and Oral Standard) and inadequate responders to biologics (ORAL Step, ORAL Sync and ORAL Solo).

Globally, the studies confirmed the clinical and radiographic efficacy of tofacitinib at both the doses of 5 and 10 mg twice daily. In the ORAL Start trial [148], the coprimary efficacy endpoints, consisting of the mean change from baseline of the modified total Sharp score (mTSS) and the ACR70 response rate at month 6, were met: of note, the mean change in the mTSS from baseline was significantly smaller in the tofacitinib groups than in the MTX group, although changes were modest in all the three groups. Significant clinical and radiographic improvements were reported as early as month 1 and sustained over 24 months.

Similarly, in the ORAL Solo, ORAL Step, ORAL Standard, and ORAL Scan trials, benefits concerning DAS28-erythrocyte sedimentation rate (ESR) scores, ACR20-ACR50-ACR70 response rates, HAQ-DI and PROs scores were already reported at month 3 [147,150,151,152].

The ORAL Standard trial, conducted on RA patients who were MTX-non responder, compared tofacitinib to adalimumab [151]. Although a formal non-inferiority comparison among the active treatments was not made, the clinical efficacy of tofacitinib resulted numerically similar to that observed with adalimumab, with clinical responses achieved in both the two treatment arms by 1 month and sustained to month 12. These results are in contrast to what was reported with baricitinib in the RA-BEAM trial, in which baricitinib showed superiority over adalimumab in the ACR20 response rate and mean change in DAS28-CRP at week 12 [119]. Although studies differed in the design, the apparent higher efficacy of baricitinib could be reconducted to its mechanism of action. Being more selective on JAK1 than tofacitinib, baricitinib strongly prevents the secretion of IL-6, and the following IL-6 blockade may play a more powerful therapeutic effect than the inhibition of TNF-α [154]. Anyway, to date, no published data on the direct comparison between baricitinib and tofacitinib are available, although a trial (ClinicalTrials.gov ID NCT03755466) is ongoing.

The phase 3b/4 ORAL Strategy trial [155], conducted in RA patients with active disease despite a previous treatment with MTX, showed comparable results in terms of ACR responses, DAS28-ESR remission and low disease activity rates at month 6 between the tofacitinib (5 mg twice a day) and the adalimumab arm. Of note, in this study, tofacitinib monotherapy showed less efficacy than in combination with MTX.

Physical function (HAQ-DI score and other PROs) improved from baseline to a generally similar extent in patients receiving tofacitinib plus MTX, adalimumab plus MTX or tofacitinib monotherapy [156].

The long-term effect of tofacitinib has been explored in the ORAL Sequel study, including 4481 patients who had previously completed a phase I, II, or III study of tofacitinib and received open-label tofacitinib 5 mg or 10 mg twice a day [87]. Statistical analysis demonstrated that both clinical and functional index scores were maintained over the time between months 1 and 96 and were generally similar with tofacitinib 5 mg and 10 mg twice a day. Also, CDAI- and SDAI-defined remission were still reported in approximately one third of patients at month 96, with a limited structural damage progression during longer-term therapy [157].

A synthesis of the main characteristics of clinical trials on tofacitinib is reported in Table 4.

### 5.3. Selected Populations

#### 5.3.1. Pediatric Patients

Evidence on the use of tofacitinib in pediatric population is limited. Few preliminary studies have been performed to establish the safety and pharmacokinetics of tofacitinib in patients affected by JIA [158]. The profile of efficacy and safety of tofacitinib 1–5 mg twice a day has been investigated in a recently completed phase III, randomized withdrawal, double-blind, placebo-controlled study in JIA patients (2 to <18 years) (A3921104; ClinicalTrials.gov ID NCT02592434; data not published). A phase II-III, long-term, open-label, follow-up study is also ongoing for those JIA patients who have previously participated in qualifying/index JIA studies of tofacitinib, including phase I studies (A3921165; Clinicaltrials.gov: NCT01500551), while a phase III, randomized, withdrawal, double blind, placebo-controlled study is currently recruiting patients with systemic JIA (A3921165; ClinicalTrials.gov ID NCT03000439).

Recently, Huang et al. [159] reported the case of a 13-year-old girl with recalcitrant systemic JIA non responder to glucocorticoids, cDMARDs and etanercept, who was prescribed with tofacitinib 2.5 mg twice daily. The authors observed a stable improvement of both articular and systemic symptoms after 2 months of treatment, with the achievement of a complete remission at month 3. Interestingly, no disease relapse or safety concerns occurred throughout the six months of follow-up.

#### 5.3.2. Selected Ethnic Groups

The efficacy and safety profile of tofacitinib has been investigated in the long-term extension study A3921041 (ClinicalTrial.gov ID: NCT00661661), conducted in 486 Japanese patients who had participated to prior phase II or phase III studies of tofacitinib as monotherapy or in combination with MTX. Final results demonstrated a sustained efficacy profile of tofacitinib (with or without MTX), consistent with that observed in the main phase III studies, along with a stable safety profile, although a higher risk of HZV reactivation has been highlighted in Japanese patients compared to the general population [153].

### 5.4. Indirect Studies Comparing Tofacitinib Efficacy

As no robust evidence is available concerning direct comparisons between biologics in RA clinical trials, indirect comparisons (network meta-analysis and registries) could provide the most relevant and comprehensive data for a relative efficacy assessment.

One systematic review and network meta-analysis assessed the safety and effectiveness of biologics (abatacept, adalimumab, anakinra, certolizumab pegol, etanercept, golimumab, infliximab, rituximab and tocilizumab) and tofacitinib in RA patients who had an inadequate response to cDMARD treatment [160]. Data, collected from 79 trials and including 32,874 participants, failed to show a superiority of mono- or combo-therapy with tofacitinib in terms of improvement in the ACR50 response compared to biologic agents. Similarly, in patients taking cDMARDs, there was no significant difference between the likelihood of having better HAQ-DI scores following the administration of biologics or tofacitinib.

Another systematic review and network meta-analysis, assessing the safety and efficacy of tofacitinib in biologic-resistant patients, included data from 12 trials extrapolated on a cohort of 3364 participants [161]. Data analysis showed that for every 100 patients treated with tofacitinib plus MTX instead than with MTX alone, 19 additional patients would experience significant improvement in their RA symptoms (based on ACR50 response) compared to 16 patients treated with a biological agent plus MTX. Similarly, it was estimated that for every 100 patients treated with tofacitinib plus MTX instead than MTX alone, 6 extra patients would achieve DAS44 or DAS28 remission compared to 10 extra patients treated with a biologic drug plus MTX.

A network meta-analysis by Vieira et al., analyzing 5 trials for a total of 2136 patients, revealed that tofacitinib at a dose of 5 mg twice a day combined with MTX was similar to biologics (abatacept, golimumab, rituximab, and tocilizumab) combined with cDMARDs in terms of the relative risk (RR) of ACR20, ACR50, and ACR70 responses and change from baseline in HAQ-DI scores [162].

These findings were confirmed by another network meta-analysis, which aimed to assess the efficacy of tofacitinib at a dose of 5 and 10 mg twice a day given either as monotherapy or combined with MTX or other cDMARDs, in comparison with biologic and synthetic therapies (abatacept, adalimumab, anakinra, certolizumab pegol, etanercept, golimumab, infliximab, tocilizumab, and baricitinib) at 24 weeks [163]. Tofacitinib, given twice a day at a dose of 5 mg, showed comparable results to those observed with the use of other biologic monotherapies (tocilizumab, certolizumab, etanercept and adalimumab) in terms of ACR20 and ACR70 response rate at week 24. In addition, the combo-therapy of tofacitinib 5 and 10 mg twice a day with MTX or other cDMARDs was more effective than certolizumab 400 mg every 4 weeks plus MTX or other cDMARDs in the achievement of the ACR70 response. Tofacitinib 10 mg twice a day revealed higher efficacy in the ACR20 response rate than etanercept, abatacept, and infliximab (all combined with cDMARDs). At 24 weeks, the ACR50 response rate indicated significantly higher efficacy of tofacitinib 5 and 10 mg twice a day compared to baricitinib (all administered with concomitant therapies). Also, a higher percentage of patients under tofacitinib reached the ACR70 response at 24 weeks compared to adalimumab, abatacept, etanercept, infliximab and baricitinib 2 mg daily plus cDMARDs.

Recently, results from the Swiss RA registry on a cohort of 2600 patients, of whom 806 treated with tofacitinib, were published [164]. The drug retention rate of tofacitinib was higher than that of anti-TNF agents but comparable with that of non-anti-TNF biologics (abatacept or anti-IL-6 agents). The use of cDMARDs improved the effectiveness of anti-TNF drugs, but not that of tofacitinib or non-anti-TNF biologics, supporting the efficacy of these drugs in monotherapy. Another recent study extrapolated real-life data from the Corrona US registry in order to evaluate the effectiveness of tofacitinib vs. anti-TNF drugs in RA patients [165]. A total of 558 subjects treated with tofacitinib from 2012 to 2016 were included. Interestingly, tofacitinib, either as mono- or combo-therapy, proved to be still effective even in patients with long-standing RA and receiving the drug as third or fourth option.

### 5.5. Safety

A safety report from phase II and III RTCs of tofacitinib showed that the majority of adverse events had a mild to moderate severity [147,148,149,150,151,152,166]. The most common side effects were nasopharyngitis (prevalence ≥ 1/10), lower respiratory tract infections, HZV reactivation, urinary tract infections, nausea, increase in creatinine serum levels and liver enzymes, dyslipidemia with a modest and reversible increase of LDL and HDL levels [167], neutropenia, anemia, oedema, headache, and dyspnea (prevalence between 1/10 and 1/100) [134].

The decrease in neutrophil count has been considered to be dose-dependent [168], and, when it occurs in a moderate manner, it may be associated with a better clinical response in RA [82]. Generally, neutrophil count is stabilized after three months since the start of a treatment with tofacitinib. Hemoglobin value has been reported to initially decrease and then to slowly increase during the treatment [87,169].

As for baricitinib, the concomitant administration of MTX may increase the risk of hyper-transaminasemia, and Japanese and Korean patients were reported to have a higher incidence of HZV infection than other populations [153].

Among SAE, the most common (prevalence between 1/10 and 1/100) were infections and malignancies (lympho-proliferative disorders and non-melanoma skin cancers) [170,171]. An integrated analysis of pooled safety data obtained from phase II and III RCTs on 5671 treated patients reported 107 malignancies developing under tofacitinib treatment [172]. Malignancies mainly affected the lungs, breast, or lymphoid organs, however they were stable over time and demonstrated an incidence in line with that reported in RA patients with a moderate to severe disease activity.

Of note, in May 2019 the European Medicines Agency (EMA)’s Safety Committee (PRAC) put a warning on the use of tofacitinib 10 mg twice a day in individuals at high risk of lung thromboembolic events. These include patients suffering from heart failure, cancer, inherited blood clotting disorders or a history of blood clots, or subjects taking combined hormonal contraceptives, hormone replacement therapy or who undergo major surgery. This warning derived from the ongoing phase IV study A3921133 (ClinicalTrials.gov ID: NCT02092467), preliminarily reporting an increased risk of pulmonary embolism and death in RA patients assuming tofacitinib 10 mg twice a day. Although this dosage is currently not recommended in RA patients in clinical practice, patients at risk of thrombotic events should be carefully monitored [173].

The most common adverse drug reactions reported by MedDRA system organ class in Eudravigilance, FAERS, and VigiAccess database are reported in Table 5. 

### 5.6. Pharmacoeconomics

A few economic evaluations were carried out in the US for tofacitinib, showing limited additional costs or even potential cost saving [174,175]. When given in monotherapy in MTX-intolerant patients, or with MTX in anti-TNF-intolerant patients, tofacitinib proved to be a less costly option compared to other bDMARDs as second-line treatment [176].

A treatment strategy with tofacitinib as either second- or third-line therapy after MTX may be a cheaper option, compared with the introduction of tofacitinib as fourth-line after cycling through 2 anti-TNF agents [177]. This finding was in line with a previous economic evaluation of tofacitinib vs. a set of biologic agents. This study showed that tofacitinib 5 mg twice a day was a cost-effective treatment option for RA compared to adalimumab or etanercept. This was due to lower costs per patient when the drug was given in monotherapy or in combination with other cDMARDs in MTX-intolerant patients. The same was observed in anti-TNF-intolerant patients, in whom tofacitinib plus MTX was more cost-effective than adalimumab plus MTX [178]. Such results were confirmed by Kulikov et al., who assessed that therapy with tofacitinib could reduce the annual cost of RA treatment from 8846€ to 2037€ per patient in comparison with other bDMARDs [176]. According to a NICE Appraisal, tofacitinib in combination with MTX is a cost-effective use of National Health Service resources in patients with severe RA with inadequate response to cDMARDs, except for etanercept biosimilar in combination with MTX [179]. In patients with severe RA with inadequate response to bDMARDs, tofacitinib in combination with MTX was more cost-effective only in the group of RTX non-eligible patients. Tofacitinib monotherapy showed a less expensive, though slightly less effective, profile than that of comparators, and its use may replace the combo-therapy with MTX in MTX-intolerant patients. In MTX-intolerant patients, tofacitinib and tocilizumab monotherapy extendedly dominated a monotherapy with adalimumab and etanercept biosimilars [179]. Another recent study indicated tofacitinib as a dominant strategy (more effective and less costly) in patients affected by moderate to severe RA who are refractory to conventional and biologic drugs in second line and third line treatments, respectively [180], compared to other alternatives.

## 6. Second Generation JAKi

Considering the FDA and EMA approval of baricitinib and tofacitinib for the treatment of adults affected by moderate to severe anti-TNF resistant RA and for the treatment of moderate to severe MTX-refractory RA, active PsA and moderate to severe anti-TNF-refractory ulcerative colitis respectively, other JAKi have been developed for RA, reaching, in two cases, the market. Among them, upadacitinib was licensed in US and Europe and peficitinib authorized in Japan for the treatment of adult patients with moderate to severe active RA and an inadequate response or intolerance to MTX, while filgotinib and decernotinib are still under clinical investigation for RA. Itacitinib, a JAKi licensed for different therapeutic indications, has also been tested in RA in a phase II RCT. Current evidence concerning these compounds is reported in the next subparagraphs.

**Figure 3:**
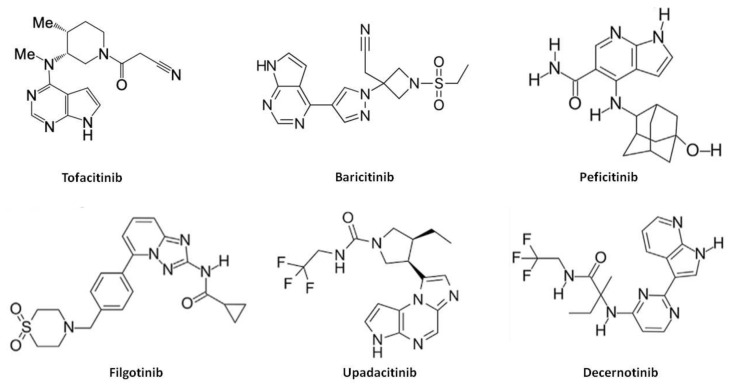
Molecular structure of first and second generation JAKi compared.

A synopsis of the chemical structure and the main pharmacological properties of the first and second generation JAKi aforementioned, are instead provided in Figure 3 and Table 6. 

### 6.1. Upadacitinib

Upadacitinib (IUPAC name: 3S,4R)-3-ethyl-4-(1,5,7,10-tetrazatricyclo[7.3.0.02,6]dodeca-2(6),3,7,9,11-pentaen-12-yl)-N-(2,2,2-trifluoroethyl)pyrrolidine-1-carboxamide) was developed as a JAK1-selective inhibitor by exploiting differences in the non-conserved domains outside the active sites of JAK1 and JAK2. In cellular assays, upadacitinib displays 60 and 100 fold selectivity for JAK1 over JAK2 and for JAK1 over JAK3, respectively [181]. The drug was approved by FDA, and, more recently, by EMA, at a dosage of 15 mg once daily, for adults with moderately to severely active RA who fail to adequately respond or are intolerant to MTX [182]. Like first generation JAKi, it may be prescribed with or without MTX.

The efficacy of upadacitinib has been evaluated in two phase II trials (BALANCE 1 and BALANCE 2) [84,183], one phase IIb/III trial (SELECT-SUNRISE) [184] and five phase III RCTs (SELECT-NEXT, SELECT-BEYOND, SELECT-MONOTHERAPY, SELECT-EARLY, SELECT-COMPARE) [185,186,187,188,189,190]. The phase III trial SELECT-CHOICE, comparing upadacitinib and abatacept in RA patients with an inadequate response to c/bDMARDs is ongoing, and results have not published yet.

Results from BALANCE 1 and BALANCE 2 [84,183], enrolling anti-TNF failing and MTX-failing patients respectively, showed rapid, dose-dependent improvements in RA signs and symptoms, with a similar safety and tolerability profile to those of other JAKi.

Phase III RCTs recruited patients with inadequate response to at least one cDMARD, including MTX (SELECT-NEXT, SELECT-MONOTHERAPY and SELECT-COMPARE) [185,187,189], and patients with inadequate response or intolerance to bDMARDs (SELECT-BEYOND) [186]. Patients were randomly assigned to received once-daily extended-release formulations of upadacitinib 15 or 30 mg or placebo for at least 12 weeks. Overall, results of these studies showed a rapid statistically significant improvement in the ACR20 response as early as week 1, and in the ACR50 and ACR70 responses from week 2 onward with upadacitinib 15 and 30 mg [185,186]. DAS28-CRP and CDAI scores were significantly improved with both the two upadacitinib doses, with 40–50% of patients achieving low disease activity by week 12. Quality of life, physical function, fatigue, severity, and duration of morning stiffness were also significantly improved in upadacitinib arms regardless of the dose. Of note, in the SELECT-BEYOND trial, recruiting 498 RA patients failing previous lines with biologics (anti-TNF and anti-IL-6R agents), the efficacy outcomes were achieved in the upadacitinib arm vs. placebo regardless of the number or kind of previously received treatments [186].

In the SELECT-MONOTHERAPY phase III RCT [187], upadacitinib monotherapy led to statistically significant improvements in clinical and functional outcomes vs. the continuation of MTX in MTX-resistant patients. A higher and statistically significant proportion of patients receiving both the two upadacitinib doses achieved DAS28-CRP low disease activity or remission compared to those assigned to MTX alone.

In the SELECT-COMPARE trial, upadacitinib, at a dose of 15 mg once daily, outperformed adalimumab in the achievement of ACR50, HAQ and DAS28-CRP responses at week 12 in MTX-refractory RA patients. Furthermore, a higher percentage of upadacitinib-assigned patients were in low disease activity or remission at week 26 compared to the adalimumab arm [189,191].

The safety profile of upadacitinib was in line with that of non-selective JAKi. Adverse events in BALANCE 1 and 2 increased in a dose-dependent manner but were mostly mild and included infections (the most common adverse events), nausea, headache, transient increase in serum transaminases and in lipid levels (both LDL and HDL with unchanged ratio) [84,183]. A dose-dependent decrease in the levels of hemoglobin (grade 3 and 4 anemia) was also noted, as well as a decrease in lymphocyte, NK cell and neutrophil count.

Serious infections occurred in the upadacitinib 30 mg arms of the SELECT-NEXT and SELECT-BEYOND trials, but none had a fatal course [185,186]. An increased incidence of HZV reactivation was observed in all the upadacitinib treatment arms across the five trials, with two serious cases in the upadacitinib 30 mg group in the SELECT-BEYOND study [186]. Two malignancies and one major MACE occurred in the upadacitinib 30 mg arm of the SELECT-NEXT trial [185], whereas four malignancies and two MACEs were reported in the SELECT-BEYOND trial in upadacitinib arms [186]. Four cases of pulmonary embolism were also reported in the SELECT-BEYOND study, but all the patients had known additional risk factors. In the SELECT-COMPARE trial an increase in serum creatine phosphokinase (CPK) was reported in subjects receiving upadacitinib but not in those treated with the active comparator [191]. Upadacitinib is metabolized by CYP enzymes, including CYP3A, but it can be safely taken with other CYP3A-metabolized drugs, including statins [192], which might be co-prescribed due to the paradoxical effect of JAKi on lipid transport. However, attention must be paid to the plausible synergistic effect of upadacitinib and statins in inducing CPK elevation and skeletal muscle damage.

### 6.2. Peficitinib

Peficitinib (IUPAC name: 4-[[(1R,3S)-5-hydroxy-2-adamantyl]amino]-1H-pyrrolo[2,3-b]pyridine-5-carboxamide) is an oral JAKi approved in March 2019 in Japan for the treatment of RA in patients who have an inadequate response to conventional therapies [193]. The drug is still under pending development in the US and in Europe.

The recommended dose of peficitinib is 150 mg or less, depending on patient’s condition, in an after meal single daily administration [194].

Peficitinib displays a less target selectivity than other second generation JAKi. Together with tofacitinib, it is, in fact, considered a pan-JAK inhibitor with a higher selectivity for JAK3 (IC50 = 710 nmol/mL) than for the other JAKs [94]. The low selectivity for JAK2 limits its effects on both red blood cells and platelets suggesting a good safety profile [195].

Findings from phase I studies on healthy volunteers demonstrated that peficitinib is rapidly absorbed in fasting condition. Tmax ranged from 1 h to 1.8 h, depending on the dose, and pharmacokinetics was not significantly influenced by renal impairment [196]. Meals, instead, delay median Tmax from 1.5 h to 4.0 h and increase peficitinib exposure (AUC) by 27% [197]. In addition, the high elimination half-life allows the administration of the drug once a day [198].

Two phase II studies, involving Japanese and non-Japanese patients, aimed to investigate the efficacy and safety of peficitinib in RA patients [198,199]. The studies showed a statistically significant improvement in the ACR20 response rate at week 12 and a similar number of adverse events compared with placebo. A third study, conducted in non-Japanese (North and Latin Americans) RA patients receiving peficitinib in combination with MTX, did not reach the prefixed endpoints due to a high rate of response in the placebo group [200].

The approval of peficitinib was based mainly on the results of two 52 week phase III studies (RAJ3 and RAJ4) [201,202], aiming to evaluate the efficacy and safety of peficitinib, at a single daily dose of 100 mg or 150 mg alone or in combination with cDMARDs, in RA patients non-responder to conventional therapies. The superiority of peficitinib over placebo was demonstrated in both RAJ3 and RAJ4 studies. At week 12, peficitinib clearly outperformed placebo in terms of ACR20 and ACR50 response rates in the RAJ3 study [201], while the ACR70 response was significantly achieved only in the 150 mg peficitinib arm. In the RAJ4 trial, a significant change from baseline in mTSS at week 28 was also reported [202].

The safety profile was in line with that of other already available JAKi and no red flags presented during these studies.

### 6.3. Filgotinib

Filgotinib (IUPAC name: N-[5-[4-[(1,1-dioxo-1,4-thiazinan-4-yl)methyl]phenyl]-[1,2,4]triazolo[1,5-a]pyridin-2-yl]cyclopropanecarboxamide) was designed following a kinase-focused-high-throughput library screening, which identified triazolopyridines as JAK-1 selective catalytic inhibitors [203]. The drug, currently under regulatory review, potently and selectively inhibits JAK1 (IC50 = 629 nmol/mL) [94]. Due to its target selectivity, filgotinib is estimated to have a good efficacy and a better safety profile than unselective JAKi. In particular, as JAK1 is not involved in the signaling pathway of erythropoietin, colony-stimulating factor, and thrombopoietin, filgotinib should not increase the risk of anemia and thrombocytopenia [204].

The efficacy and safety of filgotinib in MTX-unresponsive RA patients were tested in two exploratory phase IIa trials [205], including a monocentric four-week proof of concept study and a multicentric four-week preliminary study. In the first trial, patients were randomized to receive filgotinib 200 mg daily or placebo, whereas in the second one, they were randomly assigned to different doses of filgotinib (30 mg, 75 mg, 150 mg, or 300 mg once daily) or placebo. Both the studies evidenced a satisfactory efficacy profile of the drug. In the proof of concept study, a statistically significant number of patients achieved an ACR20 response compared to placebo. Furthermore, a reduction in serum CRP levels and in DAS28-CRP scores was also reported in the filgotinib-assigned arm. Conversely, the treatment with filgotinib was not associated with a significant improvement in the ACR20 response rate in the four-week preliminary study, although a trend was observed for the 300 mg dose. No safety issues were reported.

These initial data together with those obtained from healthy volunteers paved the way for the development of a population’s pharmacokinetic/pharmacodynamic model to be used for dose selection in phase IIb studies [206,207]. The pharmacodynamic effect of filgotinib is, in fact, given by the parent drug and its active metabolite. The latter derives from the loss of the cyclopropyl carboxylic acid group following the action of carboxylesterases [204]. Despite having a lower target selectivity for JAK1 compared to filgotinib (IC50 = 11.9 µmol/ml), the active metabolite has an elimination half-life of 23 h, allowing the administration of the compound in a single daily dose [205]. A wide dose range and different dosing regimens, with or without MTX, were investigated in 24-week dose finding phase IIb studies in patients with active RA despite the concomitant use of MTX (DARWIN 1 and DARWIN 2) [206,207]. Results showed that filgotinib at a dose of 100 or 200 mg once daily, given either as mono- or combo-therapy, was efficacious and well tolerated. Recently, the published results of the 24 week FINCH2 phase III study confirmed the efficacy of filgotinib 100 mg and 200 mg once daily in RA patients refractory to one or more bDMARD [208]. No opportunistic infections, malignancies, or fatalities were recorded during the observational period.

### 6.4. Decernotinib

Decernotinib (IUPAC name: (2R)-2-methyl-2-[[2-(1H-pyrrolo[2,3-b]pyridin-3-yl)pyrimidin-4-yl]amino]-N-(2,2,2-trifluoroethyl)butanamide) is an oral selective JAK3 inhibitor whose development for the treatment of RA is presumed to have been discontinued. JAK3 is expressed in lymphoid cells and its blockade may prevent the signaling of several cytokines involved in autoimmunity, including IL-2, IL-4, IL-7, and IL-15 [209], without affecting red blood cells and platelets.

Decernotinib was discovered following a library screening of compounds targeting JAK3 and chemically modified in order to enhance its binding-affinity and potency [210]. The drug was tested in phase IIa and IIb RCTs as monotherapy [211] or in combination with MTX [212] or other cDMARDs [213]. When given as monotherapy in RA patients with uncontrolled disease despite the use of at least 1 cDMARD, decernotinib, at dosages of 25 mg, 50 mg, 100 mg, or 150 mg twice a day, led to a significant improvement in the ACR20 response and DAS28-CRP scores at week 12 compared to placebo. Best benefits were observed in patients who received one of the 3 higher doses (50 mg, 100 mg, and 150 mg). Clinical remission, defined by a DAS28-CRP score < 2.6, was significantly greater in patients assigned to decernotinib 100 mg and 150 mg compared to placebo. The administration of decernotinib at a dose of 150 mg twice a day also resulted in better ACR50 and ACR70 response rates than those observed with placebo.

Dose titration studies showed that the ACR50 response was significantly higher with all the doses at week 12 and 24, while the achievement of the ACR70 response at week 12 was only significant with the doses of 150 mg once a day and of 100 mg twice a day [212].

Genovese et al. investigated the efficacy of the drug in the achievement of clinical and magnetic resonance imaging (MRI) outcomes in patients with inadequate DMARD response and randomly assigned to decernotinib (100 mg, 200 mg or 300 mg once daily) or placebo for 12 weeks [213]. Compared to placebo, the ACR20, DAS28-CRP and RA MRI scoring (RAMRIS) responses improved over 12 weeks across all the decernotinib dosages in a dose-dependent manner when combined with a stable cDMARD background.

The treatment with decernotinib was generally well tolerated. Adverse events were more frequent when the drug was administered at higher doses. The most common adverse events were nausea, headache, HZV reactivation, nasopharyngitis, diarrhea, upper respiratory trait infections, increased ALT serum levels, and hypercholesterolemia.

In the phase IIa RCT, the incidence of infections was similar between placebo and decernotinib assigned groups, but the frequency was higher with the 100 and 150 mg doses compared to 50 mg and 25 mg doses. Five serious infections occurred, all in the decernotinib treatment arms, with two fatalities due to pneumonia and subarachnoid hemorrhage.

Twelve of the 13 serious infections reported in the phase IIb studies (decernotinib + MTX) occurred in decernotinib-treated patients (with nine cases of pneumonia/bronchitis), and included two fatalities due to pneumonia and cardiac failure. Other adverse events encompassed increases in serum lipids and creatinine and the decrease in lymphocyte blood count, all of which were dose-dependent.

As decernotinib is a potent inhibitor of CYP3A4, this may represent a limitation in its use when coprescribed with CYP3A4-metabolized medications, like statins.

### 6.5. Itacitinib

Itacitinib (INCB039110; Incyte Corporation, Wilmington, DE) (IUPAC name: 2-[1-[1-[3-fluoro-2-(trifluoromethyl)pyridine-4-carbonyl]piperidin-4-yl]-3-[4-(7*H*-pyrrolo[2,3-d]pyrimidin-4-yl)pyrazol-1-yl]azetidin-3-yl]acetonitrile) is a novel oral selective JAK1 inhibitor, approved in 2018 by EMA as an orphan drug for the treatment of graft-versus-host disease (GVHD) [214], and currently tested for other clinical indications, including oncologic and immunologic disorders. In RA, itacitinib has been tested in a phase II randomized, dose-ranging, placebo-controlled trial [215]. The study evaluated the efficacy of itacitinib 100 mg twice a day, 200 mg twice a day, and 300 and 600 mg once daily vs. placebo in patients with active RA despite cDMARDs. The study showed that patients assigned to the highest dose had better clinical improvements and a sustained ACR20 response than patients in the other arms, regardless of background therapy or previous biologic experience. Responses were observed as early as at the first assessment time (14 days). Itacitinib was generally well tolerated. No grade 3 or grade 4 adverse events (AE) nor serious and opportunistic infections were reported. A dose-related increase in LDL was noted, though without any change in the HDL/LDL ratio. Safety data obtained from studies on GVHD patients treated with itacitinib suggest that no dose adjustment is required for a single dose of 300 mg even in patients with severe renal impairment or end-stage renal disease [216]. Furthermore, studies on healthy participants showed that single doses ranging from 10 to 1000 mg are safe from a cardiovascular point of view [217].

Nevertheless, to date, no registration trials have been designed for the use of itacitinib in RA.

## 7. Concluding Discussion

The JAK-STAT signaling pathway plays a central role in many physiopathological processes, including antimicrobial defense, hematopoiesis, post-natal growth and metabolism [71]. When activated in a canonical way, it mediates cytokine and growth factor intracellular signals; however, a non-canonical activation has also been described and associated with the epigenetic control of chromatin stability and with cell homeostasis [74]. Consequently, JAK-STAT dysregulation may be at the basis of many pathological conditions, ranging from solid and hematologic malignancies to insulin resistance, obesity and immune-mediated diseases [71,93,146,218,219]. Hence, several JAKi have been developed for the treatment of myeloproliferative and lymphoproliferative disorders, as well as for solid tumors [71].

More recently, the advent of the anti-rheumatic JAKi constituted a breakthrough in the therapeutic algorithm of RA and other inflammatory diseases, including spondyloarthritis and inflammatory bowel diseases. The panorama of JAKi is constantly enriching with new incoming molecules designed to be more specific on targeted cells and pathways, thus resulting in a better efficacy and safety profile. Compared to their biologic counterparts, JAKi differ in the route of administration, safety and efficacy profile and costs of manufacturing. Given the evidence of superiority or non-inferiority of JAKi vs. adalimumab emerging from RCTs [125,156,190], the 2020 updated EULAR therapeutic guidelines [113] recommended the use of JAKi as an alternative to biologics in RA patients refractory to cDMARDs and having poor prognostic factors, as well as in those failing a previous biologic or synthetic line [24]. A preference for biologics or JAKi should be accorded based on contraindications, monotherapy need or cost issues. Furthermore, data from RCTs evidenced a rapid and maintained efficacy of JAKi regardless of the co-prescription of MTX or other cDMARDs, and the safety profile of JAKi appeared in line with that of other authorized treatments for RA. However, some side effects, including thromboembolic events or HZV reactivation, seem to specifically occur with this class of medications. Due to a higher target selectivity, second generation JAKi may display a better safety profile.

Nevertheless, several questions remain unanswered and should be inscribed in future research agendas: (1) the identification of biomarkers (e.g., ACPA or RF positivity) predicting a better response to JAKi; (2) the duration of JAKi treatment and issues related to a dose-reduction strategy or discontinuation in patients being in low disease activity or remission; (3) the interference played, in the long term, by other CYP-metabolized drugs on JAKi pharmacokinetic profile; (4) the efficacy and safety derived from a JAKi cycling strategy and from the combination of JAKi with bDMARDs; (5) the efficacy and safety of JAKi compared to non-anti-TNF biologics. Indirect comparison data from network meta-analysis and patient cohort registries have partly addressed the latter point with regard to tofacitinib [160,161,162,163,164,165], but no evidence currently exists for other JAKi.

Growing experience on already marketed JAKi and cumulative experimental findings on novel compounds are expected to clarify many of these aspects in the next years.

## Figures and Tables

**Table 1:** Combinations of cytokines, types of JAK receptor-STAT proteins, cellular targets and possible related disorders.

Ligand	Type of Cytokine Receptor	Associated JAK Subtype	Associated STAT Subtype	Main Target Organ/Cell	Diseases Linked to an Altered Pathway	References
IL-2	Type I	JAK1 JAK3	STAT3 STAT5A/B	naïve T lymphocytes, Th1 lymphocytes	alopecia, rheumatoid arthritis, systemic lupus erythematosus, psoriasis, Crohn’s disease, ankylosing spondylitis	[38,39,40,41,42,43]
IL-3	Type I	JAK2	STAT3 STAT5A/B STAT6	Th2 lymphocytes	alopecia, rheumatoid arthritis, atopic dermatitis, systemic lupus erythematosus, ulcerative colitis	[38,39,44]
IL-4	Type I	JAK1 JAK3	STAT6	naïve T lymphocytes, Th2 lymphocytes	alopecia, rheumatoid arthritis, atopic dermatitis, systemic lupus erythematosus, psoriasis, ankylosing spondylitis, ulcerative colitis	[38,39,45]
IL-5	Type I	JAK2	STAT3 STAT5A/B STAT6	Th2 lymphocytes	alopecia, rheumatoid arthritis, atopic dermatitis, systemic lupus erythematosus, ulcerative colitis	[38,39,44]
IL-6	Type I	JAK1 JAK2 TYK2	STAT1 STAT3	naïve T lymphocytes, human keratinocytes	alopecia, rheumatoid arthritis, atopic dermatitis, systemic lupus erythematosus, ulcerative colitis, Crohn’s disease, ankylosing spondylitis, psoriasis	[11,38,39,46,47]
IL-7	Type I	JAK1 JAK3	STAT3 STAT5A/B	naïve T lymphocytes, Th1 lymphocytes	alopecia, rheumatoid arthritis, systemic lupus erythematosus, psoriasis, Crohn’s disease, ankylosing spondylitis	[38,39,40,41,42,43]
IL-9	Type I	JAK1 JAK3	STAT3 STAT5A/B	naïve T lymphocytes, Th1 lymphocytes	alopecia, rheumatoid arthritis, systemic lupus erythematosus, psoriasis, Crohn’s disease, ankylosing spondylitis	[38,39,40,41,42,43]
IL-10	Type II	JAK1 JAK2 TYK2	STAT3 STAT5A/B	Treg lymphocytes	alopecia, rheumatoid arthritis, systemic lupus erythematosus	[38,39,48]
IL-11	Type I	JAK1 JAK2 TYK2	STAT1 STAT3	naïve T lymphocytes, immunoglobulin-producing B cells, hematopoietic stem cells, megakaryocyte progenitor cells	alopecia, rheumatoid arthritis, atopic dermatitis, systemic lupus erythematosus, ulcerative colitis, Crohn’s disease, ankylosing spondylitis, anemia, leukopenia	[11,38,39,46,47]
IL-12	Type I	JAK2 TYK2	STAT4	naïve T lymphocytes	alopecia, rheumatoid arthritis, atopic dermatitis, systemic lupus erythematosus, ulcerative colitis, Crohn’s disease, ankylosing spondylitis	[11,38,39,49]
IL-13	Type I	JAK1 JAK2 JAK3 TYK2	STAT6	Th2 lymphocytes, human bronchial smooth muscle cells	alopecia, rheumatoid arthritis, atopic dermatitis, systemic lupus erythematosus, ulcerative colitis, asthma	[38,39,50]
IL-15	Type I	JAK1 JAK3	STAT3 STAT5A/B	naïve T lymphocytes, Th1 lymphocytes	alopecia, rheumatoid arthritis, systemic lupus erythematosus, psoriasis, Crohn’s disease, ankylosing spondylitis	[38,39,40,41,42,43]
IL-19	Type II	JAK1 JAK2 TYK2	STAT3	innate immune system	/	[38,39,51]
IL-20	Type II	JAK1 JAK2 TYK2	STAT3	innate immune system, human keratinocytes	psoriasis	[38,39,52,53]
IL-21	Type I	JAK1 JAK3	STAT3 STAT5A/B	naïve T lymphocytes, Th1 lymphocytes	alopecia, rheumatoid arthritis, systemic lupus erythematosus, psoriasis, Crohn’s disease, ankylosing spondylitis	[38,39,40,41,42,43,54]
IL-22	Type II	JAK1 JAK2 TYK2	STAT1 STAT3 STAT5A/B	Th17 lymphocytes	alopecia, rheumatoid arthritis, systemic lupus erythematosus, Crohn’s disease, ankylosing spondylitis, psoriasis	[38,39,53]
IL-23	Type I	TYK2 JAK2	STAT3 STAT4	naïve T lymphocytes	alopecia, rheumatoid arthritis, atopic dermatitis, systemic lupus erythematosus, ulcerative colitis, Crohn’s disease, ankylosing spondylitis	[11,38,39,49,55]
IL-24	Type II	JAK1	STAT3	immune cells, colonic epithelial cells	inflammatory bowel disease	[38,39,56]
IL-27	Type I	JAK1 JAK2 TYK2	STAT1 STAT2 STAT3 STAT4 STAT5A/B	Th1 lymphocytes, cytotoxic T cell, Treg lymphocytes	autoimmune disorders	[38,39,57]
IL-28	Type II	JAK1 TYK2	STAT1 STAT2 STAT3 STAT4 STAT5A/B	immune cells, human keratinocytes	/	[38,39,54]
IL-29	Type II	JAK1 TYK2	STAT1 STAT2 STAT3 STAT4 STAT5A/B	immune cells	/	[38,39,54]
IL-31	Type I	JAK1 JAK2	STAT1 STAT3 STAT5A/B	lung, skin, thymus, spleen, myelomonocytic cells	atopic dermatitis	[38,58]
IFN-α/β	Type II	JAK1 TYK2	STAT1 STAT2 STAT4 STAT3	naïve T lymphocytes, Th1 lymphocytes	alopecia, rheumatoid arthritis, atopic dermatitis, systemic lupus erythematosus, ulcerative colitis, Crohn’s disease, ankylosing spondylitis	[11,38,39,59,60]
IFN-γ	Type II	JAK1 TYK2	STAT1	naïve T lymphocytes, Th1 lymphocytes, human salivary glands, human keratinocytes	alopecia, rheumatoid arthritis, atopic dermatitis, systemic lupus erythematosus, ulcerative colitis, Crohn’s disease, ankylosing spondylitis, psoriasis	[38,39,61]
GM-CSF and G-CSF	Type I	JAK2	STAT3 STAT5A/B	cells belonging to the neutrophil lineage, from haemopoietic stem cells to mature neutrophils, antigen presenting cells	anemia, leukopenia, autoimmune disorders, acute myelogenous leukemia	[38,39,62,63]
GH	Type I	JAK2	STAT3 STAT5A/B	all tissues	/	[38,39,50,64,65]
EPO	Type I	JAK2	STAT5A/B	erythroid precursor cell at colony-forming units	anemia	[38,39,66]
Thrombopoietin	Type I	JAK2	STAT1 STAT3 STAT5A/B	earliest erythroid progenitors	thrombocytopenia	[38,39,67]
Leptin	Type I	JAK2	STAT3 STAT5A/B	brain, peripheral tissues	/	[38,39,68]

(JAK: Janus kinase; STAT: JAK signal transducer and activator of transcription; IL: interleukin; GM-CSF: granulocyte-macrophage colony-stimulating factor; TYK: tyrosine kinase; Th: T helper; GH: growth hormone; EPO: erythropoietin; G-CSF: granulocyte-colony stimulating factor; IFN: interferon; Treg, T regulatory).

**Table 2:** Baricitinib phase III trials in moderate to severe rheumatoid arthritis.

Study	RA-BEGIN MTX-Naïve (*n* = 588)	RA-BEAM MTX-IR (*n* = 1308)	RA-BUILD cDMARD-IR (*n* = 684)	RA-BEACON bDMARD-IR (*n* = 527)	RA-BEYOND OLE Study (*n* = 3073)
Inclusion criteria	- RA - Patients who received no prior cDMARD therapy (up to 3 weekly MTX doses permitted)	- Active RA - Patients with inadequate response to MTX, who received therapy for ≥12 weeks before trial entry, including ≥8 weeks at stable doses	- Active RA and inadequate response or intolerance to ≥ 1 cDMARD - Use of up to 2 concomitant cDMARDs was permitted at entry; these must have been used for at least 12 preceding weeks with stable doses for at least 8 preceding weeks	- Moderately to severe active RA - Patients must have previously received ≥ 1 TNFi and discontinued the treatment because of an inadequate response or unacceptable side effects - bDMARDs must have been discontinued at least 4 weeks before randomization (≥6 months for rituximab) - Use of ≥1 concomitant cDMARD at entry; these must have been used for at least 12 preceding weeks with stable doses for at least 8 preceding weeks	- Patients who completed a BARI phase II or phase III trial
Type of therapy	Monotherapy + combination therapy	Combination therapy	Combination therapy	Combination therapy	Monotherapy—patients who completed previous BARI RA studies
Background treatment	None/MTX	MTX	cDMARDs	cDMARDs	cDMARDs
Active comparator	MTX	ADA + MTX			
Arms	(1) BARI 4 mg sid (2) BARI 4 mg sid + MTX (3) MTX 10 mg/week	(1) PBO (2) BARI 4 mg sid (3) ADA 40 mg/sc q2wk	(1) BARI 2 mg sid (2) BARI 4 mg sid (3) PBO	(1) BARI 2 mg sid (2) BARI 4 mg sid (3) PBO	(1) BARI 2 mg sid (2) BARI 4 mg sid
Duration (weeks)	52	52	24	24	Ongoing (completion estimated in 2024)
Primary endpoint	ACR20 (Week 24)	ACR20 (Week 12)	ACR20 (Week 12)	ACR20 (Week 12)	Long term Safety
Key secondary endpoint	Week 24: DAS28-CRP HAQ-DI mTSS SDAI remission	Week 12: DAS28-CRP HAQ-DI mTSS (Week 24) SDAI remission Morning Joint stiffness	Week 12: DAS28-CRP HAQ-DI SDAI remission Morning Joint stiffness	Week 12: DAS28-CRP HAQ-DI SDAI remission	Long term Efficacy
Main results (ACR20):	(Week 24) BARI 4 mg vs. MTX: 77% vs. 62% (*p* ≤ 0.01); BARI 4 mg vs. BARI 4 mg + MTX: 77% vs. 78% (Week 52) BARI 4 mg vs. MTX: 73% vs. 56% (*p* ≤ 0.05); BARI 4 mg vs. BARI 4 mg + MTX: 73% vs. 73%	(Week 12) BARI vs. PBO: 70% vs. 40% (*p* < 0.001); BARI vs. ADA: 70% vs. 61% (*p* = 0.014) (week 24) BARI vs. PBO: 74% vs. 37% (*p* < 0.001); BARI vs. ADA: 74% vs. 66% (*p* ≤ 0.05)	(Week 12) BARI 2 mg vs. PBO: 66% vs. 39% (*p* ≤ 0.001); BARI 4 mg vs. PBO: 62% vs. 39% (*p* ≤ 0.001)	(Week 12) BARI 2 mg vs. PBO: 49% vs. 27% (*p* < 0.001); BARI 4 mg vs. PBO: 55% vs. 27% (*p* < 0.001) (Week 24) BARI 2 mg vs. PBO: 45% vs. 27% (*p* ≤ 0.001); BARI 4 mg vs. PBO: 46% vs. 27% (*p* ≤ 0.001)	Currently recruiting

The table summarizes the design and outcomes of baricitinib phase III confirmatory studies for rheumatoid arthritis patients (RA: rheumatoid arthritis; ACR: American College of Rheumatology; ACR20: improvement by 20% from baseline of core set parameters; ADA: adalimumab; BARI: baricitinib; cDMARD: conventional disease-modifying anti-rheumatic drug; HAQ-DI: health assessment questionnaire-disability index; MTX: methotrexate; OLE: open label extension; mTSS: modified total Sharp score; PBO: placebo; SDAI: Ssimplified disease activity index; TNFi: tumor necrosis factor inhibitor; DAS28-CRP: disease activity score on 28 joints by C-reactive protein; sc q2w: subcutaneous injection every other week; sid: once a day).

**Table 3:** List of adverse reactions reported under treatment with baricitinib by MedDRA system organ class in Eudravigilance, FAERS and Vigiacess database.

	Number of Individual Cases by Reaction Groups (Updated on 28 February 2020)		
Reaction Groups	VigiAccess	Eudravigilance	FAERS
Blood and lymphatic system disorders	176 (2.0%)	98 (3.5%)	46 (2.2%)
Cardiac disorders	117 (1.3%)	57 (2.1%)	54 (2.6%)
Congenital, familial and genetic disorders	1 (0.0%)	1(0.0%)	2 (0.1%)
Ear and labyrinth disorders	41 (0.5%)	15 (0.5%)	11 (0.5%)
Endocrine disorders	7 (0.1%)	1 (0.0%)	1 (0.0%)
Eye disorders	110 (1.3%)	36 (1.3%)	32 (1.6%)
Gastrointestinal disorders	879 (10.0%)	310 (11.2%)	181(8.8%)
General disorders and administration site conditions	1308 (14.9%)	326 (11.8%)	282(13.7%)
Hepatobiliary disorders	66 (0.8%)	42 (1.5%)	46 (2.2%)
Immune system disorders	88 (1.0%)	15 (0.5%)	26 (1.3%)
Infections and infestations	2095 (23.9%)	615 (22.2%)	371 (18.0%)
Injury, poisoning and procedural complications	348 (4.0%)	77 (2.8%)	91(4.4%)
Metabolism and nutrition disorders	162 (1.8%)	75 (2.7%)	28 (1.4%)
Musculoskeletal and connective tissue disorders	889 (10%)	151 (5.4%)	129 (6.3%)
Neoplasms benign, malignant and unspecified (including cyst and polyps)	99 (1.1%)	70 (2.5%)	100 (4.9%)
Nervous system disorders	566 (6.5%)	175 (6.3%)	158 (7.7%)
Pregnancy, puerperium and perinatal conditions	4 (0.0%)	4 (0.1%)	1 (0.0%)
Product issues	3 (0.0%)	0 (0.0%)	3 (0.1%)
Psychiatric disorders	206 (2.3%)	56 (2.0%)	47 (2.3%)
Renal and urinary disorders	134 (1.5%)	40 (1.4%)	37 (1.8%)
Reproductive system and breast disorders	49 (0.6%)	16 (0.6%)	5 (0.2%)
Respiratory, thoracic and mediastinal disorders	612 (7%)	247 (8.9%)	170 (8.3%)
Skin and subcutaneous tissue disorders	504 (5.7%)	199 (7.2%)	89 (4.3%)
Surgical and medical procedures	60 (0.7%)	1 (0.0%)	73 (3.5%)
Vascular disorders	248 (2.8%)	147 (5.3%)	75 (3.6%)
Total	8772 (100%)	2774 (100%)	2058 (100%)

**Table 4:** Tofacitinib phase III clinical trials in moderate to severe rheumatoid arthritis.

Trial	ORAL Start MTX-naïve (*n* = 958)	ORAL Solo c/bDMARD-IR (*n* = 611)	ORAL Sync c/bDMARD-IR (*n* = 795)	ORAL Scan MTX-IR (*n* = 797)	ORAL Standard MTX-IR (*n* = 717)	ORAL Strategy MTX-IR (*n* = 1146)	ORAL Step TNF-IR (*n* = 399)
Participants	MTX-naïve patients with active RA	Active RA patients with inadequate response to ≥ 1 c/bDMARD receiving stable doses of antimalarial	Active RA patients with inadequate response to ≥ 1 c/bDMARD	Active RA patients receiving background MTX	Active RA patients receiving stable doses of MTX	Active RA patients receiving stable doses of MTX	Moderate to severe RA patients with inadequate response to anti-TNF drugs
Type of therapy	Monotherapy	Monotherapy	Combination therapy	Combination therapy	Combination therapy	Monotherapy	Combination therapy
Active Comparator	MTX	/	/	/	ADA	ADA + MTX non-inferiority	/
Background treatment	None	None	cDMARD	MTX	MTX	None	MTX
Arms	(1) TOFA 5 mg bid (2) TOFA 10 mg bid (3) MTX	(1) TOFA 5 mg bid (2) TOFA 10 mg bid (3) PBO advanced at 3 months to TOFA 5 mg bid or 10 mg bid	(1) TOFA 5 mg bid (2) TOFA 10 mg bid (3) PBO advanced to TOFA 5 mg bid or 10 mg bid at 6 months (3 months for non-responders)	(1) TOFA 5 mg bid (2) TOFA 10 mg bid (3) PBO advanced to TOFA 5 mg bid or 10 mg bid at 6 months (3 months for non-responders)	(1) TOFA 5 mg bid (2) TOFA 10 mg bid (3) PBO advanced to TOFA 5 mg bid or 10 mg bid at 6 months (3 months for non-responders) (4) ADA	(1) TOFA 5 mg bid (2) TOFA 10 mg bid (3) ADA + MTX	(1) TOFA 5 mg bid (2) TOFA 10 mg bid (3) PBO advanced to TOFA 5 mg bid or 10 mg bid at 3 months
Duration (months)	24	6	12	24	12	12	6
Features	X-Rays			X-Rays			
Coprimary endpoints	∆mTSS ACR70 (month 6)	ACR20 HAQ-DI DAS28-ESR < 2.6 (month 3)	ACR20 DAS28-ESR < 2.6 (month 6) HAQ-DI (month 3)	ACR20 ∆mTSS DAS28-ESR < 2.6 (month 6) HAQ-DI (month 3)	ACR20 DAS28-(ESR) < 2.6 (month 6) HAQ-DI (month 3)	ACR50 (month 6)	ACR20 HAQ-DI DAS28-ESR < 2.6 (month 3)
Main results	(Month 6) ACR20 (% pts): 71.3 (*p* < 0.001) TOFA 5 mg; 76.1 (*p* ≤ 0.01) TOFA 10 mg; 50.5 MTX. ACR70 (% pts): 25.5 (*p* < 0.001) TOFA 5 mg; 37.7 (*p* ≤ 0.01) TOFA 10 mg; 12 MTX. HAQ-DI (change from BL): −0.8 (*p* < 0.001) TOFA 5 mg; −0.9 (*p* < 0.001) TOFA 10 mg; −0.6 MTX. DAS28-ESR < 2.6 (% pts): 14.6 (*p* ≤ 0.05) TOFA 5 mg; 21.8 (*p* ≤ 0.01) TOFA 10 mg; 7.6 MTX. ∆mTSS (from baseline): 0.2 (*p* < 0.001) TOFA 5 mg; <0.1 (*p* < 0.001) TOFA 10 mg; 0.8 MTX	(Month 3) ACR20 (% pts): 59.8 (*p* < 0.001) TOFA 5 mg; 65.7 (*p* < 0.001) TOFA 10 mg; 26.7 PBO. HAQ-DI (change from BL): −0.5 (*p* < 0.001) TOFA 5 mg; −0.57 (*p* < 0.001) TOFA 10 mg; −0.19 PBO. DAS28-ESR < 2.6 (% pts): 5.6 TOFA 5 mg; 8.7 TOFA 10 mg; 4.4 PBO	(Month 6) ACR20 (%pts): 52.7 (*p* < 0.001) TOFA 5 mg+ cDMARD; 56.6 (*p* < 0.001) TOFA 10 mg + cDMARD; 31.2 PBO + cDMARD. HAQ-DI (change from BL): −0.44 (*p* < 0.001) TOFA 5 mg + cDMARD; −0.53 (*p* < 0.001) TOFA 10 mg+ cDMARD; −0.21 PBO + cDMARD. DAS28-ESR < 2.6 (% pts): 8.5 (*p* ≤ 0.01) TOFA 5 mg + cDMARD; 12.5 (*p* < 0.001) TOFA 10 mg + cDMARD; 2.7 PBO + cDMARD	(Month 6) ACR20 (%pts): 51.5 (*p* < 0.001) TOFA 5 mg + MTX; 61.8 (*p* < 0.001) TOFA 10 mg + MTX; 25.3 PBO + MTX. DAS28-ESR < 2.6 (% pts): 7.2 TOFA 5 mg + MTX; 16.0 (*p* < 0.001) TOFA 10 mg + MTX; 1.6 PBO + MTX. HAQ-DI (change from BL): −0.40 TOFA 5 mg + MTX; −0.54 (*p* < 0.001) TOFA 10 mg + MTX; −0.15 PBO + MTX. ∆mTSS (from baseline): 0.12 TOFA 5 mg + MTX; 0.06 (*p* ≤ 0.05) TOFA 10 mg + MTX; 0.47 PBO + MTX	(Month 6) ACR20 (%pts): 51.5 (*p* < 0.001) TOFA 5 mg + MTX; 52.6 (*p* < 0.001) TOFA 10 mg + MTX; 47.2 (*p* < 0.001) ADA + MTX; 28.3 PBO+ MTX. HAQ-DI (change from BL): −0.55 (*p* < 0.001) TOFA 5 mg+ MTX; −0.61 (*p* < 0.001) TOFA 10 mg + MTX; −0.49 (*p* < 0.001) ADA + MTX; −0.24 PBO + MTX. DAS28-ESR < 2.6 (% pts): 7 (*p* ≤ 0.05) TOFA 5 mg + MTX; 12.5 (*p* < 0.001) TOFA 10 mg + MTX; 6.7 (*p* ≤ 0.05) ADA + MTX; 1.1 PBO + MTX	(Month 6) ACR20 (% pts): 65 TOFA 5 mg; 73.1 TOFA 5 mg + MTX; 71 ADA+ MTX. ACR50 (% pts): 38.3 TOFA 5 mg; 46 TOFA 5 mg + MTX; 44 ADA+ MTX. HAQ-DI (change from BL): −0.54 TOFA 5 mg; −0.59 TOFA 5 mg + MTX; −0.54 ADA + MTX. DAS28-ESR < 2.6 (% pts): 10.4 TOFA 5 mg; 12 TOFA 5 mg + MTX; 12.4 ADA + MTX.	(Month 3) ACR20 (% pts): 41.7 (*p* ≤ 0.01)) TOFA 5 mg + MTX; 48.1 (*p* < 0.001) TOFA 10 mg + MTX; 24.4 PBO. + MTX HAQ-DI (change from BL): −0.43 (*p* < 0.001) TOFA 5 mg + MTX; −0.46 (*p* < 0.001) TOFA 10 mg + MTX; −0.18 PBO + MTX. DAS28-ESR < 2.6 (% pts): 6.7 (*p* ≤ 0.05) TOFA 5 mg + MTX; 8.8 (*p* ≤ 0.05) TOFA 10 mg + MTX; 1.7 PBO + MTX

The table summarizes the design and outcomes of tofacitinib phase III confirmatory studies for rheumatoid arthritis patients (RA, rheumatoid arthritis; ACR: American College of Rheumatology; ACR20: improvement by 20% from baseline of core set parameters; ADA: adalimumab; cDMARD: conventional disease-modifying anti-rheumatic drug; HAQ-DI: health assessment questionnaire-dDisability index; MTX: methotrexate; OLE: open label extension; mTSS: modified total Sharp score; PBO: placebo; SDAI: Simplified Disease Activity Index; TOFA: tofacitinib; DAS28-ESR: disease activity score on 28 joints by erythrocyte sedimentation rate; BL: baseline; bid: twice a day).

**Table 5:** List of adverse reactions reported under treatment with tofacitinib by MedDRA system organ class in Eudravigilance, FAERS and Vigiacess database.

	Number of Individual Cases by Reaction Groups (Updated on 28 February 2020)		
Reaction Groups	VigiAccess	Eudravigilance	FAERS
Blood and lymphatic system disorders	959 (0.8%)	749 (1.3%)	1276 (1.0%)
Cardiac disorders	1302(1.1%)	1240 (2.1%)	1870 (1.4%)
Congenital, familial and genetic disorders	51(0.0%)	51 (0.1%)	91 (0.1%)
Ear and labyrinth disorders	792 (0.7%)	442 (0.8%)	985 (0.7%)
Endocrine disorders	157 (0.1%)	145 (0.2%)	262 (0.2%)
Eye disorders	1681 (1.4%)	1173 (2.0%)	2176 (1.6%)
Gastrointestinal disorders	10,951 (9.4%)	4894 (8.4%)	12,200 (9.1%)
General disorders and administration site conditions	29,273 (25.2%)	10,533 (18.0%)	31,152 (23.2%)
Hepatobiliary disorders	595 (0.5%)	537 (0.9%)	939 (0.7%)
Immune system disorders	1809 (1.6%)	1052 (1.8%)	2560 (1.9%)
Infections and infestations	16,042 (13.8%)	8940 (15.3%)	16,908 (12.6%)
Injury, poisoning and procedural complications	8189 (7.0%)	5028 (8.6%)	10,407 (7.8%)
Metabolism and nutrition disorders	1385 (1.2%)	980 (1.7%)	1728 (1.3%)
Musculoskeletal and connective tissue disorders	12,602 (10.8%)	6497 (11.1%)	15271 (11.4%)
Neoplasms benign, malignant and unspecified (including cyst and polyps)	1587 (1.4%)	1611 (2.8%)	2358 (1.8%)
Nervous system disorders	8910 (7.7%)	4252 (7.3%)	9929 (7.4%)
Pregnancy, puerperium and perinatal conditions	29 (0.0%)	33 (0.1%)	59 (0.0%)
Product issues	86 (0.1%)	49 (0.1%)	112 (0.1%)
Psychiatric disorders	3026 (2.6%)	1502 (2.6%)	3567 (2.7%)
Renal and urinary disorders	1508 (1.3%)	1077 (1.8%)	1869 (1.4%)
Reproductive system and breast disorders	405 (0.3%)	223 (0.4%)	460 (0.3%)
Respiratory, thoracic and mediastinal disorders	6594 (5.7%)	3465 (5.9%)	7725 (5.8%)
Skin and subcutaneous tissue disorders	5706 (4.9%)	2306 (3.9%)	6684 (5.0%)
Surgical and medical procedures	865 (0.7%)	216 (0.4%)	1289 (1.0%)
Vascular disorders	1832 (1.6%)	1516 (2.6%)	2403 (1.8%)
Total	116,336 (100%)	58,511 (100%)	134,280 (100%)

**Table 6:** Synopsis of the main pharmacological properties of first and second generation JAKi. (JAKi, Janus kinase inhibitors; JAK, Janus kinase; TYK2, tyrosin kinase 2; sid, single dose; bid, twice daily; PK: pharmacokinetics; PD: pharmacodynamics; FDA: Food and Drug Administration; EMA: European Medicines Agency; Tmax: time to peak; bid: twice a day; sid; once a day; OAT: ornithine aminotransferase; CYP: cytochrome p450; US, United States; Eu, Europe; AUC, area under the curve; IC50, half maximal inhibitory concentration).

Drug	First generation JAKi		Second Generation JAKi			
active principle	Baricitinib	Tofacitinib	Upadacitinib	Peficitinib	Filgotinib	Decernotinib
brand name	Olumiant^®^	Xeljanz^®^	Rinvoq™	Smyraf^®^	/	/
other name	INCB028050 LY3009104	CP-690,550	ABT-494	ASP015K, JNJ-54781532	GLPG0634/GS-6034	VX-509
target	JAK1 JAK2	JAK1 JAK3 JAK2 TYK2	JAK1	JAK3 JAK1	JAK1	JAK3
Dose	2 mg sid	5 mg bid	15 mg sid	150 mg sid or 100 mg depending on the patient’s condition	100 mg or 200 mg sid	50–150 mg bid
renal insufficiency	1 mg once daily in patients with creatinine clearance between 30 and 60 mL/min Not recommended in patients with creatinine clearance < 30 mL/min	No dose adjustment in patients with mild (50–80 mL/min) or moderate (30–49 mL/min) renal impairment 5 mg once daily in patient with severe renal impairment (<30 mL/min)	No dose adjustment in patients with mild, moderate or severe renal impairment Not tested in subjects with end stage renal disease	No dose adjustment required in patients with renal impairment	/	/
liver failure	No dose adjustment in patients with mild or moderate hepatic impairment Not recommended in patients with severe hepatic impairment	No dose adjustment in patients with mild (Child Pugh A) hepatic impairment 5 mg once daily recommended in patient with moderate hepatic function (<Child Pugh B) Not recommended for use in patients with severe hepatic function (Child Pugh C)	No dose adjustment in patients with mild (Child Pugh A) or moderate (Child Pugh B) hepatic impairment Not recommended in patients with severe hepatic impairment (Child Pugh C)	50 mg sid in patients with moderate liver dysfunction Contraindicated in patient with severe liver dysfunction	/	/
development stage for rheumatoid arthritis	Approved in ∼50 countries	Approved in ∼80 countries	Approved in US and Eu	Approved in Japan Under regulatory review in South Korea and Taiwan Under clinical development in China	Under regulatory review is US, EU and Japan	Development discontinued
approval	FDA approval: yes (2018) EMA approval: yes (2017)	FDA approval: yes (2012) EMA approval: yes (2017)	FDA approval: yes (2019) EMA approval: yes (2019)	FDA approval: no EMA approval: no Japan approval: yes (2019)	FDA approval: no EMA approval: no	FDA approval: no EMA approval: no
safety	Most frequent: throat and nose infections; herpes simplex virus infection; infections causing a sick stomach or diarrhea; urinary infections; pneumonia; thrombocytosis nausea; Uncommon: leukopenia; increase in serum creatine kinase; high serum levels of triglycerides; acne; weight gain	Most frequent: upper and lower airway infections; influenza; herpes zoster virus infection; urinary tract infections; abdominal pain; vomiting; diarrhea; nausea; gastritis; rash; headache; anemia; leukopenia; hyper-transaminasemia Uncommon: tuberculosis; diverticulitis; pyelonephritis; cellulitis; herpes simplex virus infection; viral gastroenteritis and other viral infections; blood creatinine increase; blood cholesterol increase; low density lipoprotein increase; weight increase Rare: sepsis; disseminated tuberculosis involving bones and other organs; other unusual infections; joint infection	Most frequent: upper respiratory tract infections (common cold, sinus infections); nausea; cough; fever Uncommon: serious infections; malignancies; thrombosis; gastrointestinal perforations; altered laboratory parameters; embryo-fetal toxicity Rare: major adverse cardiac events pulmonary embolism; venous thromboembolism	Most frequent: nasopharyngitis; herpes zoster virus infection; blood creatine kinase increase; lymphopenia Uncommon: pneumonia; pharyngitis; epipharyngitis; upper respiratory tract infections bronchitis; influenza; cystitis Rare: sepsis; gastrointestinal perforation	Most frequent: nasopharyngitis; nausea; bronchitis; headache; upper respiratory tract infection Uncommon: major adverse cardiac events pulmonary embolism; herpes zoster virus infection; deep vein thrombosis	Most frequent: nausea; headache; nasopharyngitis; diarrhea; upper respiratory tract infections; blood cholesterol increase; alanine aminotransferase increase Uncommon: serious infections
Licensed therapeutic indications	rheumatoid arthritis	rheumatoid arthritis, psoriatic arthritis, ulcerative colitis	rheumatoid arthritis	rheumatoid arthritis	Under regulatory review for rheumatoid arthritis	
PK	Tmax 1.5 h; t1/2 12.5 h	Tmax 0.5–1 h; t1/2 3.3 h	Tmax 2–4 h; t1/2 8-14 h	Tmax 1.1–2.1 h; t1/2 9.9–16.2 h (after multiple 30–200 mg twice daily dosages)	Tmax 0,5–3 h; t1/2 3.82–10.9 h (after single/multiple twice daily 25–450 mg dosages) Tmax 1–2; t1/2 (after single/multiple twice daily 30–300 mg dosages)	N.A.
IC50	IC50JAK1 5.9 nM; IC50JAK2 5.7 nM; IC50JAK3 420 nM; IC50TYK2 60 nM	IC50JAK1 3.2 nM; IC50JAK2 4.1 nM; IC50JAK3 1.6 nM; IC50TYK2 34 nM	IC50JAK1 45 nM; IC50JAK2 109 nM; IC50JAK3 2.1 μM; IC50TYK2 4.7 µM	IC50JAK1 3.9 nM; IC50JAK2 5.0 nM; IC50JAK3 0.71 nM; IC50Tyk2 4.8 nM	IC50JAK1 10 nM; IC50JAK2 28 nM; IC50JAK3 810 nM; IC50Tyk2 110 nM	IC50JAK1 10 nM; IC50JAK2 10 nM; IC50JAK3 2.5 nM; IC50TYK2 10 nM
drug interactions	OAT3 inhibitors and CYP3A4 inhibitors (e.g., ketoconazole) and inducers (e.g., rifampicin)	CYP3A4 inhibitors (e.g., ketoconazole), medicinal products that result in both moderate inhibition of CYP3A4 as well as potent inhibition of CYP2C19 (e.g., fluconazole) and CYP inducers	CYP3A4 inhibitors (e.g., ketoconazole) and inducers (e.g., rifampicin)	Verapamil (P-glycoprotein inhibitor that increase AUC and Cmax of peficitinib by 27–39%) No clinically significant interaction with methotrexate and rosuvastatin	/	CYP3A4 inhibitors
				/	/	/
